# Novel insights into the genetically obese (*ob/ob*) and diabetic (*db/db*) mice: two sides of the same coin

**DOI:** 10.1186/s40168-021-01097-8

**Published:** 2021-06-28

**Authors:** Francesco Suriano, Sara Vieira-Silva, Gwen Falony, Martin Roumain, Adrien Paquot, Rudy Pelicaen, Marion Régnier, Nathalie M. Delzenne, Jeroen Raes, Giulio G. Muccioli, Matthias Van Hul, Patrice D. Cani

**Affiliations:** 1 Metabolism and Nutrition Research group, Louvain Drug Research Institute (LDRI), Walloon Excellence in Life Sciences and BIOtechnology (WELBIO), UCLouvain, Université catholique de Louvain, Av. E. Mounier, 73 B1.73.11, 1200 Brussels, Belgium; 2 Department of Microbiology and Immunology, Rega Institute for Medical Research, VIB Center for Microbiology, KU Leuven, University of Leuven, Leuven, Belgium; 3 Bioanalysis and Pharmacology of Bioactive Lipids Research Group, Louvain Drug Research Institute (LDRI), UCLouvain, Université catholique de Louvain, Brussels, Belgium

**Keywords:** Leptin-deficiency, Leptin-receptor deficiency, *ob/ob*, *db/db*, Lipopolysaccharides, Bile acids, Liver inflammation, Adipose tissue inflammation, Short-chain fatty acids, Mouse gut microbiota, Quantitative microbiota profile

## Abstract

**Background:**

Leptin-deficient *ob/ob* mice and leptin receptor-deficient *db/db* mice are commonly used mice models mimicking the conditions of obesity and type 2 diabetes development. However, although *ob/ob* and *db/db* mice are similarly gaining weight and developing massive obesity, *db/db* mice are more diabetic than *ob/ob* mice. It remains still unclear why targeting the same pathway—leptin signaling—leads to the development of two different phenotypes. Given that gut microbes dialogue with the host via different metabolites (e.g., short-chain fatty acids) but also contribute to the regulation of bile acids metabolism, we investigated whether inflammatory markers, bacterial components, bile acids, short-chain fatty acids, and gut microbes could contribute to explain the specific phenotype discriminating the onset of an obese and/or a diabetic state in *ob/ob* and *db/db* mice.

**Results:**

Six-week-old *ob/ob* and *db/db* mice were followed for 7 weeks; they had comparable body weight, fat mass, and lean mass gain, confirming their severely obese status. However, as expected, the glucose metabolism and the glucose-induced insulin secretion were significantly different between *ob/ob* and *db/db* mice. Strikingly, the fat distribution was different, with *db/db* mice having more subcutaneous and *ob/ob* mice having more epididymal fat. In addition, liver steatosis was more pronounced in the *ob/ob* mice than in *db/db* mice. We also found very distinct inflammatory profiles between *ob/ob* and *db/db* mice, with a more pronounced inflammatory tone in the liver for *ob/ob* mice as compared to a higher inflammatory tone in the (subcutaneous) adipose tissue for *db/db* mice. When analyzing the gut microbiota composition, we found that the quantity of 19 microbial taxa was in some way affected by the genotype. Furthermore, we also show that serum LPS concentration, hepatic bile acid content, and cecal short-chain fatty acid profiles were differently affected by the two genotypes.

**Conclusion:**

Taken together, our results elucidate potential mechanisms implicated in the development of an obese or a diabetic state in two genetic models characterized by an altered leptin signaling. We propose that these differences could be linked to specific inflammatory tones, serum LPS concentration, bile acid metabolism, short-chain fatty acid profile, and gut microbiota composition.

**Video abstract.**

**Supplementary Information:**

The online version contains supplementary material available at 10.1186/s40168-021-01097-8.

## Background

Over the past 40 years, obesity has reached epidemic proportions and has become a huge public health and economic issue since it is a major contributor to several metabolic comorbidities, including insulin resistance, type 2 diabetes (T2D), and liver diseases [1–3]. Obesity is characterized by an imbalance between energy intake and energy expenditure [4, 5], although its prevalence within individuals varies with behavior, genetic, environmental, and physiological factors [6]. It is well established that obesity is associated with a state of chronic, low-grade inflammation distinguished by the production of several inflammatory cytokines and adipokines [7]. In the last two decades, the gut microbiota has emerged as a fundamental environmental factor modulating whole-body metabolism by influencing energy balance, glucose metabolism, gut barrier function, and low-grade inflammation among others [8]. Numerous metabolic functions can be traced back to microbial metabolites, of which the short-chain fatty acids (SCFAs) are the most studied and have been associated with several metabolic functions [9, 10]. Moreover, the gut microbiota has been shown to modulate the bile acid (BA) profile, mainly by metabolizing primary BA into secondary BA, thus increasing their chemical diversity. These molecules are also known for regulating several host metabolic processes [11].

Obesity is a risk factor in which several organs and systems are involved. Among these, the liver and adipose tissue play a central role. Contrary to the metabolic function of the liver, the adipose tissue has the capacity to store and release energy under the form of lipids as well as the ability to act as an active endocrine organ capable of synthesizing a wide variety of biologically active compounds (i.e., adipokines) that are involved in the regulation of several metabolic pathways [12]. The best-known adipokine is leptin, which is mainly produced by mature adipocytes. Besides its role in satiety, leptin plays an important role in the regulation of energy homeostasis, lipid and glucose metabolism, and the immune response via the cognate leptin receptor (ObR) [13, 14]. Alterations in leptin signaling are closely associated with metabolic diseases, such as obesity and T2D [15]. The genetic leptin-deficient *ob/ob* mice and the leptin-resistant *db/db* mice are therefore widely used as animal models to study obesity and related metabolic disorders [16–19]. *Ob/ob* mice are characterized by a mutation of the obese (*ob*) gene encoding leptin, whereas the *db/db* mice have a mutation of the diabetes (*db*) gene encoding for the ObR [20]. Both mouse models have defective leptin signaling with a complete lack of leptin production in *ob/ob* mice and an overexpression of circulating leptin in *db/db* mice to which they cannot respond due to a complete deficiency of the long isoform of the leptin receptor (ObRb) [15]. Despite a different underlying molecular mechanism at the base of the leptin deficiency (ligand versus receptor), both models show a similar phenotype in regard to hyperphagia, hypometabolism, and obesity, but manifest different impairments in glucose metabolism [20, 21]. Indeed, the *ob/ob* mice develop obesity and mild insulin resistance, while the *db/db* mice develop obesity and diabetes. These differences are not yet fully understood as many mechanistic details associating leptin signaling with the development of an obese and a diabetic state remain poorly investigated. Recent studies using both genetic models have identified novel markers of obesity and T2D [18], as well as a different gut microbiota composition across different ages that were closely linked to fluctuations in blood glucose [22]. However, identification of novel mediators and a better understanding of the different metabolic pathways associated with the leptin signaling could result in the development of new potential therapeutic strategies to tackle obesity and its related metabolic disorders. This study aimed at explaining why despite having the same fat mass and the same body weight, the onset of metabolic complications observed in both *ob/ob* and *db/db* mice matched by age and sex and fed an identical diet for 7 weeks were different. To explore this hypothesis, we have characterized inflammatory markers, bacterial components, BA, SCFAs, and gut microbes.

## Methods

### Mice and experimental design

Male homozygous *ob/ob* mice (B6.V-Lepob/ob/JRj) were used as a leptin-deficient obese model, and their lean littermates served as controls (CT ob); (n = 9–10 per group). Male homozygous *db/db* mice (BKS-Lepr/db/db/JOrlRj), functionally deficient for the long-form leptin receptor, were used as a hyperleptinemic obese type 2 diabetic model, and their lean littermates served as controls (CT db); (n = 9–10 per group). Mice were purchased at the same time and from the same supplier (Janvier Laboratories, Le Genest-Saint-Isle, France) at the age of 6 weeks. Mice were housed in a specific pathogen- and opportunistic-free (SOPF) controlled environment (room temperature of 22 ± 2 °C, humidity 55 ± 10%, 12 h daylight cycle, lights off at 6 p.m.) in groups of two mice per cage, with free access to sterile food and sterile water. Upon delivery, mice underwent an acclimation period of one week, during which they were fed a standard diet containing 10% calories from fat (D12450Ji; Research Diet; New Brunswick, NJ, USA) and were then kept ad libitum on the same diet for 7 weeks. Milli-Q water filtered by a Millipak® Express 40 with a 0.22-μm membrane filter (Merck Millipore, Burlington, Massachusetts, USA) was autoclaved and provided ad libitum. All mouse experiments were approved by and performed in accordance with the guideline of the local ethics committee (Ethics committee of the Université catholique de Louvain for Animal Experiments specifically approved this study that received the agreement number 2017/UCL/MD/005). Housing conditions were specified by the Belgian Law of 29 May 2013, regarding the protection of laboratory animals (agreement number LA1230314).

### Measurements during the study

Body weight, food, and water intake were recorded three times per week. Body composition was assessed weekly by using 7.5-MHz time domain-nuclear magnetic resonance (TD-NMR) (LF50 Minispec; Bruker; Rheinstetten, Germany).

### Oral glucose tolerance test and insulin resistance index

In the 6th week of the experiment, mice were fasted for 6 h and given an oral glucose load (1 g glucose per kg body weight). Blood glucose was measured 30 min before oral glucose load (− 30 min) and 15, 30, 60, 90, and 120 min after oral glucose load. Blood glucose was determined with a glucose meter (Accu Check, Roche, Basel, Switzerland) on blood samples collected from the tip of the tail vein.

Plasma insulin concentration was determined on blood samples using an ELISA kit (Mercodia, Uppsala, Sweden) according to the manufacturer’s instructions. Insulin resistance index was determined by multiplying the area under the curve of both blood glucose (− 30 to 120 min) and plasma insulin (− 30 and 15 min) obtained following the oral glucose tolerance test.

### Collection of fecal material

For microbial composition analysis, freshly defecated feces were collected after the acclimation period (day 0), after 3 weeks (day 21), and after 6 weeks (day 42) and kept on dry ice before storage at − 80 °C. In order to determine the fecal energy contents, fecal samples were collected in the 5th week of the experiment during a 24-h period after mice were transferred to clean cages. The samples were dried overnight at 60 °C and weighted to assess the amount of feces secreted per day. Then energy content was measured on a C1 calorimeter from IKA (Germany). Per cage containing two animals, one mean value was considered for analysis.

### Tissue sampling

At the end of the experimental period and after 6 h of fasting, mice were anesthetized with isoflurane (Forene, Abbott, Queenborough, Kent, UK). Portal vein blood was collected in a lipopolysaccharide (LPS) free tube, while vena cava blood was collected in EDTA-containing tubes. After centrifugation (12 000×*g* for 5 min) serum and plasma were aliquoted and immediately immersed in liquid nitrogen before storage at − 80 °C for further analysis. Liver, brown and white adipose tissues (subcutaneous, epididymal, and visceral), muscles (soleus, gastrocnemius, tibialis, and vastus lateralis), and cecal content were precisely dissected, weighed, and immediately snap-frozen in liquid nitrogen and stored at − 80 °C for further analysis.

### Histological analysis and immunohistochemistry

A portion of the liver and subcutaneous adipose tissue (SAT) were fixed in 4% paraformaldehyde solution for 24 h at room temperature. Samples were then immersed in ethanol 100% for 24 h before processing for paraffin embedding and preparation of 5-μm tissue sections. Adipocyte size was determined on H&E stained sections and macrophage infiltration was quantified after immunostaining with F4/80 antibody (Ab6640, Abcam, Cambridge, UK). Images were captured at × 20 magnification and obtained using a SNC400 slide scanner and digital Image Hub software 561 (Leica Biosystems, Wetzlar, Germany). Analyses were performed using ImageJ (version 1.48r, National Institutes of Health, Bethesda, Maryland, USA) in a blinded manner. Crown-like structures (CLSs) were counted both in the hepatic and adipose tissue as an indicator of immune cell recruitment and inflammation and were expressed as the number of CLSs per field. A minimum of 5 high-magnification fields were analyzed per mouse.

### RNA preparation and real-time qPCR analysis

Total RNA was prepared from collected tissues using TriPure reagent (Roche). Quantification and integrity analysis of total RNA was performed by running 1 μl of each sample on an Agilent 2100 Bioanalyzer (Agilent RNA 6000 Nano Kit, Agilent, Santa Clara, CA, USA). cDNA was prepared by reverse transcription of 1 μg total RNA using a Reverse Transcription System Kit (Promega, Madison, Wisconsin, USA). Real-time PCR was performed with the CFX96 Real-time PCR system and CFX manager 3.1 software (BioRad, Hercules, California, USA) using GoTaq qPCR Master Mix (Promega, Madison, Wisconsin, USA) for detection, according to the manufacturer’s instructions. *RPL19* RNA was chosen as the housekeeping gene, and data were analyzed according to the 2^−ΔΔCT^ method. The identity and purity of the amplified product were assessed by melting curve analysis at the end of amplification. The primer sequences for the targeted mouse genes are presented in the Additional file 1: Table S1.

### Biochemical analyses

Total lipids were measured after extraction with chloroform-methanol according to a modified Folch method [23] as previously described [24]. Triglyceride and cholesterol concentrations were measured using a kit coupling an enzymatic reaction and spectrophotometric detection of the final product (Diasys Diagnostic and systems, Holzheim, Germany). All analyses and samples were run in duplicate.

### Lipopolysaccharides assay

LPS levels were measured in serum collected from the portal vein of *ob/ob*, *db/db*, and their respective lean littermates using a competitive inhibition enzyme immunoassay (Cloud-Clone Corp, Houston, TX). Samples were diluted (1:10) with the Charles River Endosafe dispersing agent (Charleston, South Carolina, USA) to disperse endotoxin molecules during sample preparation, and heated 15 min at 70 °C to inactivate nonspecific inhibitors of endotoxin. Samples displaying hemolysis were excluded from the analysis according to the manufacturer’s instructions. The endotoxin concentration was determined spectrophotometrically at 450 nm and calculated from the standard curve of known amounts of *Escherichia coli* endotoxin. All determinations were performed in duplicate.

### Bile acid and short-chain fatty acid quantification

Bile acids and SCFAs were quantified using an HPLC-MS adapted method, as previously described [25]. Briefly, for BA analysis, liver tissue was homogenized in ice-cold distilled water and proteins precipitated using acetone (in the presence of 7 deuterated internal standards). Next, samples were centrifuged, supernatants recovered, and evaporated to dryness. Chromatographic separation was achieved using an Ascentis Express C-18 column (100 × 4.6 mm, 2.7 μm) (Sigma-Aldrich) and a gradient of water and acetonitrile in the presence of formic acid. For ionization, an ESI probe operating in negative mode was used.

For SCFAs analysis, the cecal content (50–60 mg wet material) was homogenized in water followed by sonication in an ice water bath. Acetonitrile was used for protein precipitation (in the presence of valproic acid as internal standard). Following centrifugation, the supernatant was recovered and a derivatization step (using 3-nitrophenylhydrazine in the presence of EDC and pyridine) performed. Samples were purified using liquid-liquid extraction to remove the remaining reagents. After evaporation, the final residue was analyzed using an LTQ Orbitrap XL mass spectrometer coupled to an Accela HPLC system (ThermoFisher Scientific). A Hypersil GOLD PFP (100 × 2.1 mm; 1.9 μm) column using a gradient of water-acetonitrile-acetic acid and acetonitrile-acetic acid allowed separating the different isomers. For ionization, an APCI probe was used in positive mode. Calibration curves were prepared using the same conditions to determine sample content. Xcalibur® software was used for data analysis. For each cecal content, an aliquot was freeze-dried to determine a dry residue that was used for data normalization.

For both types of analytes, calibration curves were prepared using the same conditions to determine sample content. Xcalibur® software was used for data analysis.

### Microbial load measurement

Microbial load measurement by flow cytometry was determined in the fecal samples of both *ob/ob* and *db/db* mice and their littermate counterparts. Briefly, 20 mg frozen (− 80 °C) aliquots were dissolved in physiological solution to a total volume of 100 ml (8.5 g × l^−1^ NaCl; VWR International). Subsequently, the slurry was diluted 500 times. Samples were filtered using a sterile syringe filter (pore size of 5 μm; Sartorius Stedim Biotech). Next, 1 ml of the microbial cell suspension obtained was stained with 1 μl SYBR Green I (1:100 dilution in dimethylsulfoxide; shaded for 15 min of incubation at 37 °C; 10,000 concentrate, Thermo Fisher Scientific). The flow cytometry analysis was performed using a C6 Accuri flow cytometer (BD Biosciences) based on a previously published study [26]. Fluorescence events were monitored using the FL1 533/30-nm and FL3 > 670-nm optical detectors. In addition, forward- and sideward-scattered light was collected. The BD Accuri CFlow software was used to gate and separate the microbial fluorescence events on the FL1/FL3 density plot from background events. A threshold value of 2,000 was applied on the FL1 channel. The gated fluorescence events were evaluated on the forward and sideward density plot, as to exclude remaining background events. Instrument and gating settings were kept identical for all samples (fixed staining/gating strategy) [26]. On the basis of the exact weight of the aliquots analyzed, cell counts were converted to microbial loads per gram of fecal material.

### Fecal microbiota sequencing

Fecal DNA extraction and microbiota profiling by 16S rRNA gene sequencing were performed as described previously [27]. Briefly, DNA was extracted from frozen fecal pellets using the MoBio PowerMicrobiome RNA isolation kit with the addition of 10 min incubation at 90 °C after the initial vortex step. The V4 region of the 16S rRNA gene was amplified with primer pair 515F/806R. Samples were processed for multiplex sequencing with dual-index barcoding. Sequencing was performed on the Illumina MiSeq platform (San Diego, California, USA), to generate paired-end reads of 250 bases in length in each direction. After de-multiplexing using LotuS (version 1.565) [28], fastq sequencing files were pre-processed using the DADA2 pipeline (R package version 1.6.0) [29], for trimming, quality control, merging of pairs, and taxonomic annotation using the SILVA (version 132n) database [30]. With one sample failing sequencing quality control (N < 500 reads after QC), 112 fecal sequencing profiles were obtained.

### Deriving quantitative microbiota profiles

The quantitative microbiome profiling (QMP) matrix was built as described previously [31] by combining sequencing data and microbial load assessment by flow cytometry. A script is available at https://github.com/raeslab/QMP/blob/master/QMP.R. In short, samples were downsized to even sampling depth, defined as the ratio between sampling size (16S rRNA gene copy number corrected sequencing depth) and microbial load (average total cell count per gram of frozen fecal material). 16S rRNA gene copy number corrections were based on the ribosomal RNA operon copy number database rrnDB [32]. The copy number corrected sequencing depth of each sample was rarefied to the level necessary to equate the minimum observed sampling depth in the cohort (original sampling depth range = [4e−8,7e−7]). The minimum rarefaction level was 609 cnv-corrected reads (approx. 2500 non-corrected reads). The obtained rarefied-to-even-sampling-depth genus-level matrix was then converted into numbers of cells per gram. From an input of 112 samples with 101 genera (observed with minimum 1 read), with a 17-fold difference in original sampling depth, the obtained QMP matrix had a final size of 112 samples and 94 observed genera characterized at a final sampling depth of 4.11e−08 cnv-corrected reads per cell in a gram of sample. Zero values in the microbiota matrix are therefore interpretable as non-detectable genera at the final sampling depth.

## Statistical analysis

### Metabolic parameter correlation analysis

**Fig. 1:**
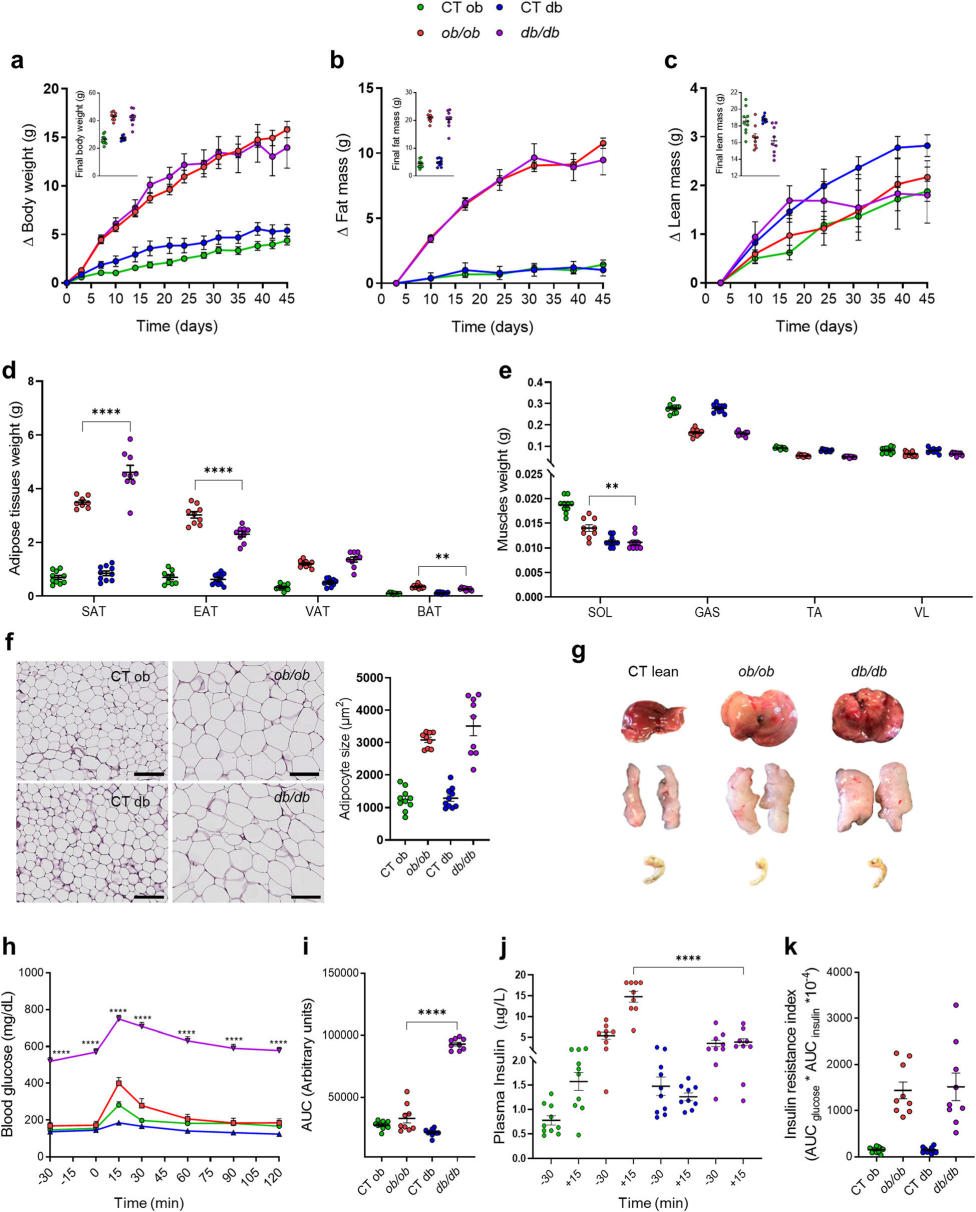
Different phenotype features between *ob/ob* and *db/db* mice*.* (**a**) ∆ (Delta) of the body weight (starting at day 0) and final body weight (g). (**b**) ∆ of the fat mass (starting at day 3) and final fat mass (g) measured by time-domain nuclear magnetic resonance (TD-NMR). (**c**) ∆ of the lean mass (starting at day 3) and final lean mass (g) measured by time-domain nuclear magnetic resonance (TD-NMR). (**d**) Adipose tissues (SAT: subcutaneous; EAT: epididymal; VAT: visceral; BAT: brown) weight (g). (**e**) Muscles (SOL: soleus; GAS: gastrocnemius; TA: tibialis; VL: vastus lateralis) weight (g). (**f**) Size of the adipocytes in the subcutaneous adipose tissue (SAT). Scale bar, 100 μm; magnification, × 20. (**g**) Morphology of the liver, SAT, and cecum. (**h**) Plasma glucose (mg/dL) profile after 1 g/kg glucose oral challenge in freely moving mice and (**i**) the mean area under the curve (AUC) measured between 0 and 120 min after glucose loading. (**j**) Plasma insulin (μg/L) measured 30 min before and 15 min after glucose loading. (**k**) Insulin resistance index determined by multiplying the AUC of blood glucose by the AUC of insulin. Green: CT ob lean mice, red: *ob/ob* mice, blue CT db lean mice, and violet: *db/db* mice. Data are presented as the mean ± s.e.m, **P < 0.01, ****P < 0.0001 (n = 8–10). Data were analyzed using two-way ANOVA followed by Tukey’s post hoc test for (**a–c)** and (**h)** and according to one-way ANOVA followed by Tukey’s post hoc test for (**d**–**f**) and (**i**–**k**)

**Fig. 2:**
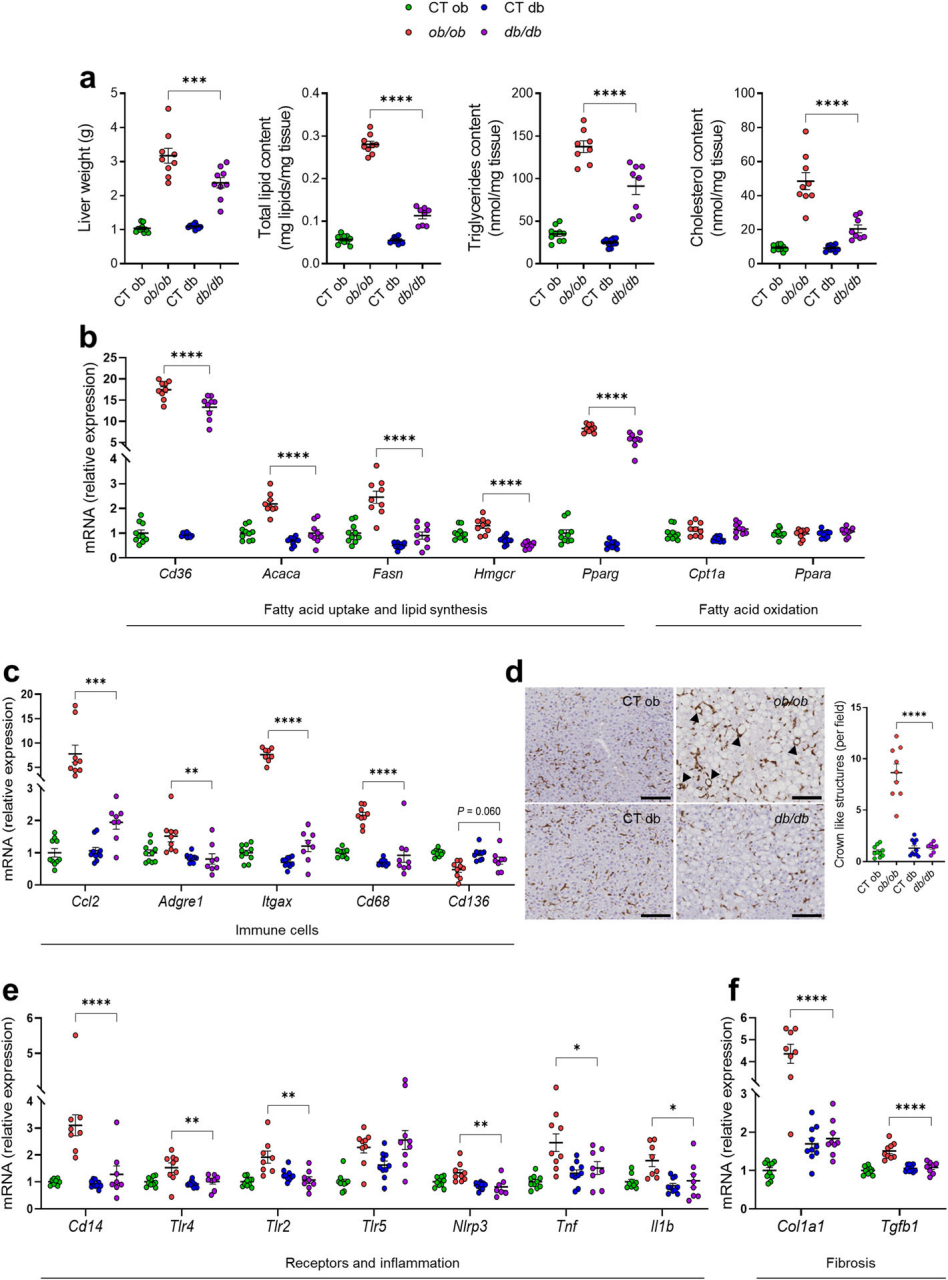
Different hepatic features between *ob/ob* and *db/db* mice. (**a**) Liver weight at necropsy (g); Total lipid content (mg lipids/mg tissue); Liver triglycerides (nmol/mg tissue); Liver cholesterol (nmol/mg tissue) measured using a spectrophotometer. (**b**) mRNA expression of liver lipid metabolism markers measured by RT-qPCR. (**c**) mRNA expression of liver immune cells markers measured by RT-qPCR. (**d**) Representative pictures of staining for F4/80 in the liver. Scale bar, 100 μm; magnification, × 20. Arrowheads point to crown-like structures. (**e**) mRNA expression of liver receptors and inflammatory cytokines markers measured by RT-qPCR. (**f**) mRNA expression of liver fibrosis markers measured by RT-qPCR. Green: CT ob lean mice, red: *ob/ob* mice, blue CT db lean mice, and violet: *db/db* mice. Data are presented as the mean ± s.e.m, *P < 0.05, **P < 0.01, ***P < 0.001, ****P < 0.0001 (n = 7–10). For the mRNA expression, relative units were calculated versus the mean of the CT ob mice values set at 1. Data were analyzed by one-way ANOVA followed by Tukey’s post hoc test

**Fig. 3:**
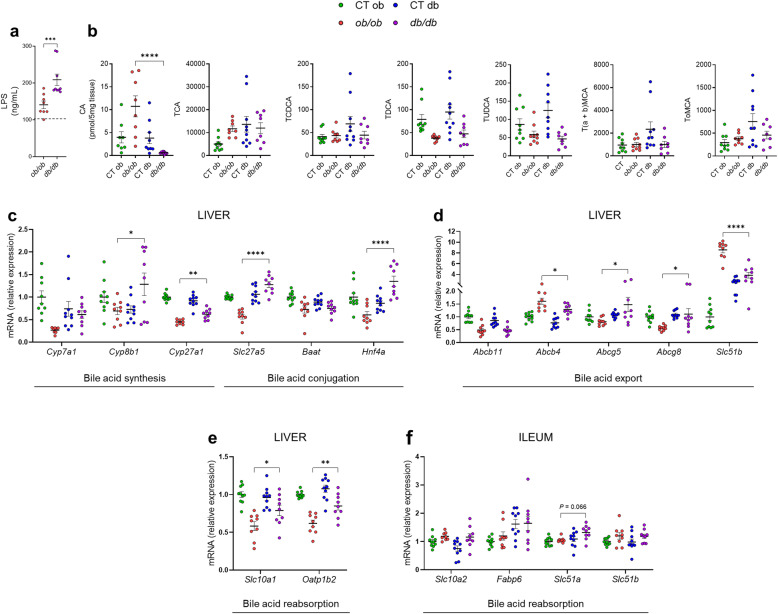
Different serum LPS concentration, hepatic bile acid content, and bile acid metabolism between *ob/ob* and *db/db* mice. (**a**) Serum LPS concentration (ng/mL) measured by competitive inhibition enzyme immunoassay. (**b**) Liver bile acid content (pmol/5mg tissue) quantified by HPLC-MS. (**c**) mRNA expression of liver bile acid synthesis and conjugation markers measured by RT-qPCR. (**d**) mRNA expression of liver bile acid export markers measured by RT-qPCR. (**e**) mRNA expression of liver bile acid reabsorption markers measured by RT-qPCR. (**f**) mRNA expression of ileal bile acid reabsorption markers measured by RT-qPCR. Dashed black line: CT lean mice, green: CT ob lean mice, red: *ob/ob* mice, blue: CT db lean mice, and violet: *db/db* mice. Data are presented as the mean ± s.e.m, *P < 0.05, **P < 0.01, ***P < 0.001, ****P < 0.0001(n = 8–10). For the mRNA expression, relative units were calculated versus the mean of the CT ob mice values set at 1. Data were analyzed by one-way ANOVA followed by Tukey’s post hoc test. CA, cholic acid; CDCA, chenodeoxycholic acid; DCA, deoxycholic acid; MCA, muricholic acid; T, taurine; UDCA, ursodeoxycholic Acid. a, alpha; b, beta; o, omega conjugated species

**Fig. 4:**
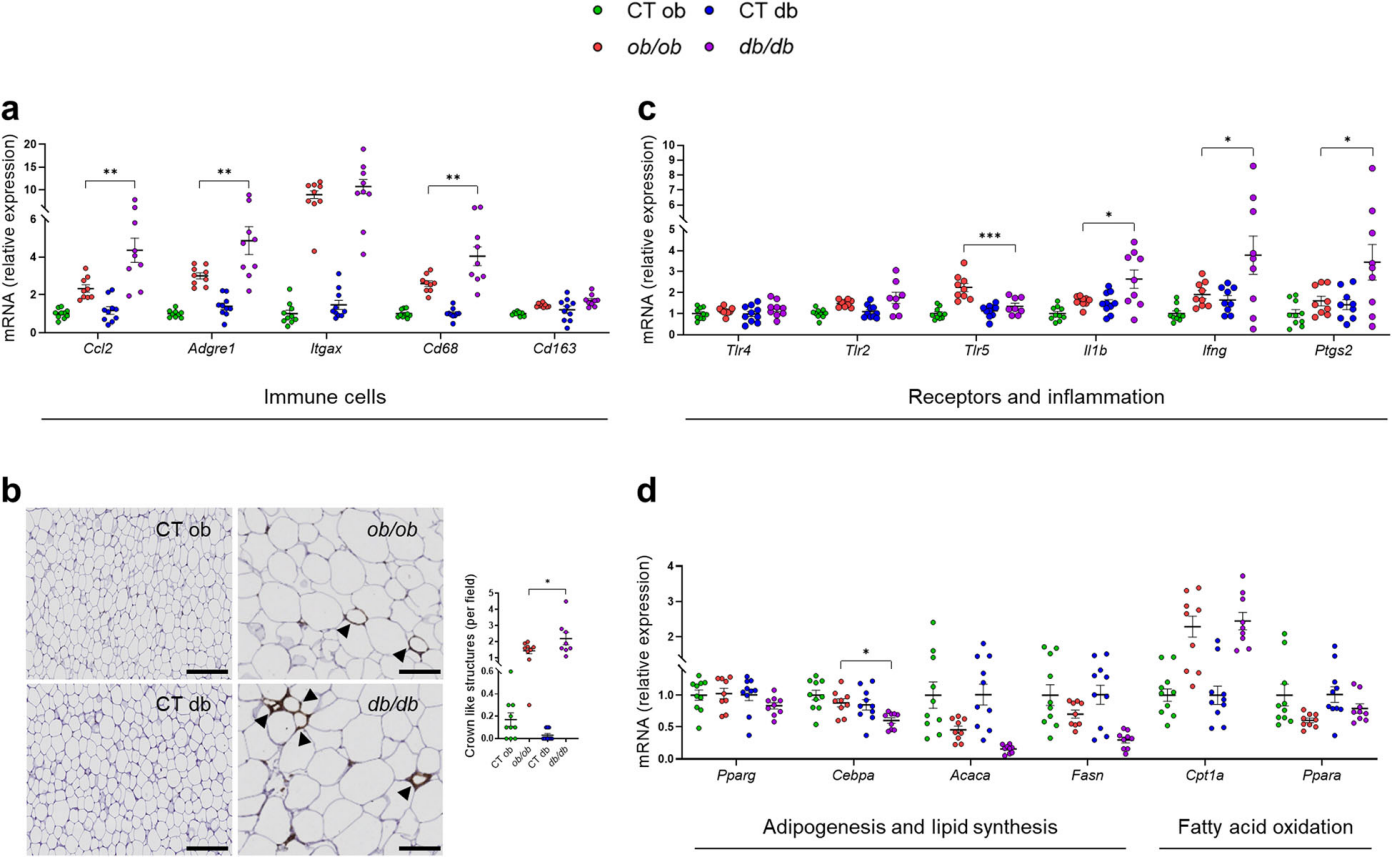
Different subcutaneous adipose tissue features between *ob/ob* and *db/db* mice. (**a**) mRNA expression of SAT immune cells markers measured by RT-qPCR. (**b**) Representative pictures of F4/80 staining in SAT. Scale bar, 100 μm; magnification, × 20. Arrowheads point to crown-like structures. (**c**) mRNA expression of SAT receptors and inflammatory cytokines markers measured by RT-qPCR. (**d**) mRNA expression of SAT lipid metabolism and adipogenesis markers measured by RT-qPCR. Green: CT ob lean mice, red: *ob/ob* mice, blue CT db lean mice, and violet: *db/db* mice. Data are presented as the mean ± s.e.m, *P < 0.05, **P < 0.01, ***P < 0.001 (n = 8–10). For the mRNA expression, relative units were calculated versus the mean of the CT ob mice values set at 1. Data were analyzed by one-way ANOVA followed by Tukey’s post hoc test

**Fig. 5:**
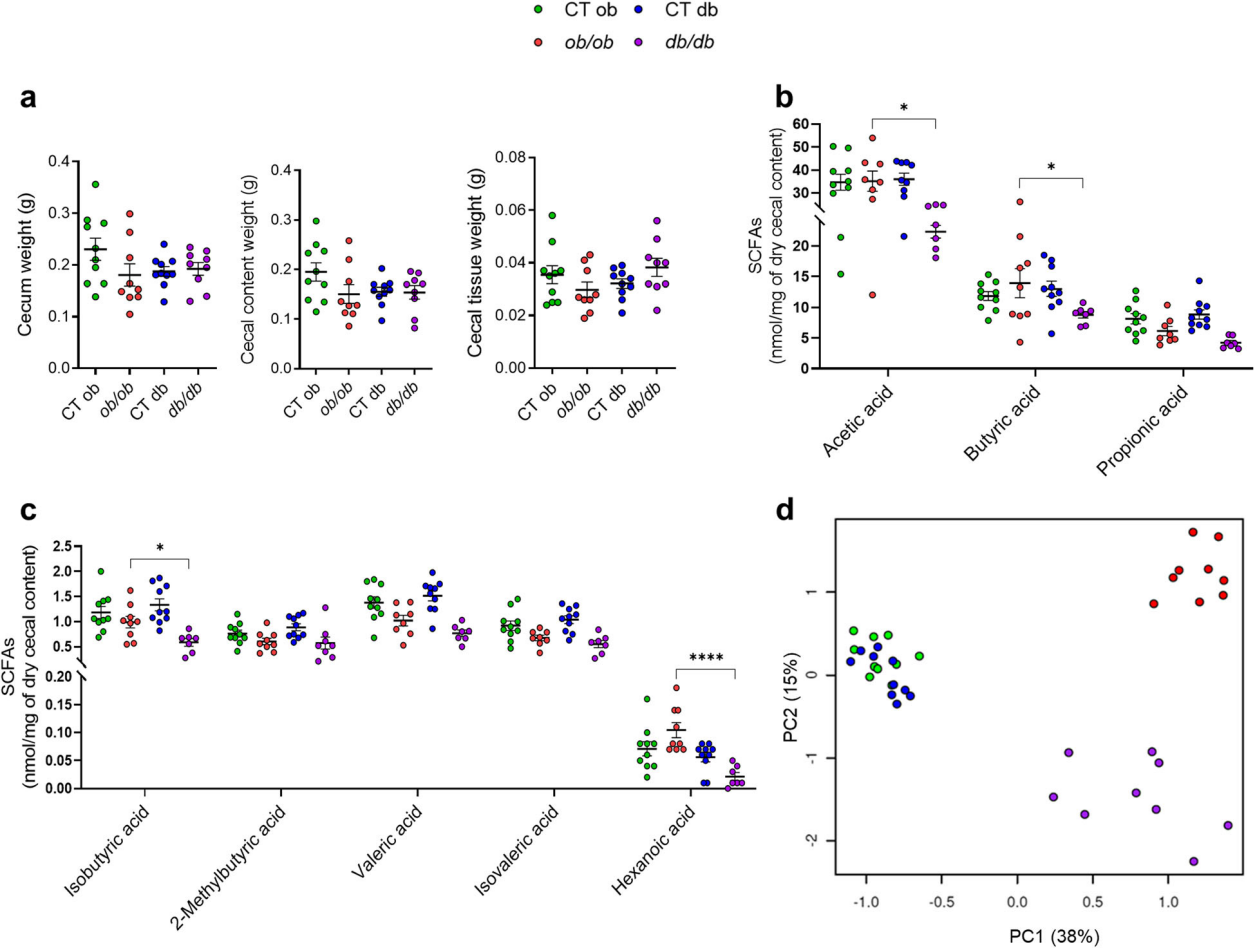
Different short-chain fatty acids profile between *ob/ob* and *db/db* mice. (**a**) Cecum weight (g); Cecal content weight (g); Cecal tissue weight (g). (**b**) Amount of acetic acid, butyric acid, and propionic acid in the cecal content (nmol/mg of dry cecal content) measured by liquid chromatography-mass spectrometry (UPLC-MS). (**c**) Amount of isobutyric acid, 2-methylbutyric acid, valeric acid, isovaleric acid, and hexanoic acid in the cecal content (nmol/mg of dry cecal content) measured by liquid chromatography-mass spectrometry (UPLC-MS). (**d**) Principal component analysis (PCA) score plot of mice based on all measured metabolic parameters. Green: CT ob lean mice, red: *ob/ob* mice, blue CT db lean mice, and violet: *db/db* mice. Data are presented as the mean ± s.e.m, *P < 0.05, ****P < 0.0001 (n = 7–10). Data were analyzed by one-way ANOVA followed by Tukey’s post hoc test for (**a–c**)

Principal component analysis (PCA) of the metabolic parameters measured in the figures (i.e., Figs. 1, 2, 3, 4, 5, and S2) of the present study was performed using the R package “psych” (version 2.0.12) [33]. Missing data (2%) was imputed using the median metabolic parameter value to be able to compute the component scores. Three principal components were extracted, following results obtained by parallel analysis (scree plot). The PCA was performed without rotation. The loadings matrix of the PCA was investigated manually to identify contrasting signs of the correlations of the variables with the principal components.

### Metabolic and fecal data association to genotype

The data are presented as the means ± s.e.m (standard error of mean). The statistical significance of difference for the metabolic parameters was evaluated by one-way or two-way ANOVA followed by Tukey’s post hoc multiple comparison test, while for the microbial load and the bacterial genera abundances, non-parametric equivalents: Kruskal-Wallis test with Dunn’s multiple comparison test, were used. For the metabolic parameters, only statistically significant differences between *ob/ob* and *db/db* mice were reported. The data with a superscript symbol (^#^ CT ob vs CT db; * *ob/ob* vs *db/db)* are significantly different (^#,^ *P < 0.05; ^##,^ **P < 0.01; ^###,^ ***P < 0.001; ^####,^ ****P < 0.0001). All the analyses were performed using GraphPad Prism version 8.00 for Windows (GraphPad Software). The presence of outliers was assessed using the Grubbs test.

### Partitioning of microbiota variation according to genotype and sampling day

Visualization of fecal microbiota profile variation was performed by principal coordinates analysis (PCoA) using Bray-Curtis dissimilarity between genus-level quantitative microbiota profiles using the R package vegan [34]. Visualization (arrows) of the direction and degree of association of mouse genotypes on microbiota composition was performed by post hoc fit on the PCoA (R package vegan *envfit* function). The explanatory power of mouse genotype and day of sampling, on microbial community genus-level QMP variation, was estimated by permutational multivariate analysis of variance (Adonis test, R package vegan *adonis2* function).

### Taxa-metabolic parameters associations

Correlations between single taxa quantitative abundances (genera) and metabolic parameters were assessed by non-parametric Spearman correlation, excluding taxa with less than 15% prevalence in the dataset.

All tests were subjected to multiple testing corrections (Benjamini-Hochberg method) whenever applicable.

## Results

### Different phenotypic features between *ob/ob* and *db/db* mice

After 7 weeks of follow-up, both *ob/ob* and *db/db* mice gradually gained the same body weight while feeding ad libitum on normal diet, thereby confirming the obesogenic effect of impaired leptin-signaling (Fig. 1a). Body composition analysis using NMR showed a similar increase in fat mass (Fig. 1b) and a lower lean mass (Fig. 1c) in both *ob/ob* and *db/db* mice. Interestingly, despite having similar total fat mass gain, at the end of the experiment, we found that both *ob/ob* and *db/db* mice had a different fat mass distribution of various fat depots. Both epididymal adipose tissue (EAT) and brown adipose tissue (BAT) showed significantly higher weight in *ob/ob* mice (23.7% and 24.7%, respectively) (Fig. 1d), whereas subcutaneous adipose tissue (SAT) was 22.9% heavier in *db/db* mice compared with *ob/ob* mice (Fig. 1d). No differences were observed for the visceral adipose tissue (VAT) mass when comparing *ob/ob* and *db/db* mice (Fig. 1d). Among the different types of muscles, the soleus (SOL) mass was the only one to have a significant 20.6% reduction in *db/db* mice compared with *ob/ob* mice (Fig. 1e). The increase in fat mass was associated with larger adipocytes in both mutant mice (Fig. 1f). During the necropsy, we also found that the morphology of different tissues (i.e., liver, adipose tissues, and cecum) in term of size, shape, and color was similar between the two control lean groups, while it was different between *ob/ob* and *db/db* mice (Fig. 1g). Despite their equal body weight and fat mass gain, *db/db* mice had an enhanced food and water intake throughout the duration of the experiment (Additional file 2: Fig. S1a-b). Measurement of body temperature showed a markedly lower temperature (− 1.2 °C) in *db/db* mice when compared to *ob/ob* mice, indicating a different energy metabolism (Additional file 2: Fig. S1c). Conversely, calculating the energy excretion (i.e. amount of feces secreted in 24h multiplied by the fecal energy content measured by bomb calorimeter) revealed that *db/db* mice had a lower energy uptake compared to *ob/ob* mice (Additional file 2: Fig. S1d-f).

### Different glucose and insulin profile between *ob/ob* and *db/db* mice

The blood glucose profile and the glucose-induced insulin secretion were significantly different between *ob/ob* and *db/db* mice. At basal levels and after the oral glucose load, fasted *db/db* mice exhibited a more pronounced impaired glucose tolerance, which was maintained throughout the duration of the oral glucose tolerance test (OGTT) as indicated by a 64.5% increase in the area under the curve (Fig. 1h, i), and a 73.9% reduction in plasma insulin levels compared with fasted *ob/ob* mice (Fig. 1j). Contrarily to an impaired insulin secretion in *db/db* mice, *ob/ob* mice produced significantly more insulin in response to oral glucose administration, suggesting an insulin resistance state (Fig. 1j). Overall, both models developed insulin resistance to a similar degree as evidenced by the calculation of the insulin resistance index (Fig. 1k).

### Different lipid and inflammatory hepatic profile between *ob/ob* and *db/db* mice

We found that *ob/ob* mice had a significant 25.1% increase in the liver weight (Fig. 2a) and displayed more severe hepatic steatosis compared to *db/db* mice. Hepatic lipid accumulation was confirmed by a 59.8% increase in total hepatic lipid contents and was mainly due to strongly increased hepatic levels of triglycerides and cholesterol (33.8% and 57.9%, respectively) (Fig. 2a). In order to understand the underlying mechanism of the development of hepatic steatosis, we analyzed a large panel of genes involved in lipid metabolism (Fig. 2b). In *ob/ob* mice, we observed a significantly higher mRNA expression of a marker linked to fatty acid uptake and storage (i.e., cluster of differentiation 36, encoded by *Cd36*). Consistent with their higher lipid and cholesterol accumulation, *ob/ob* mice displayed increased lipid synthesis markers (i.e., acetyl-CoA carboxylase alpha, encoded by *Acaca*; fatty acid synthase, encoded by *Fasn*; 3-hydroxy-3-methylglutaryl-CoA reductase, encoded by *Hmgcr*; and peroxisome proliferator-activated receptor gamma, encoded by *Pparg*) as compared to *db/db* mice, strongly suggesting a different hepatic lipid metabolism between the two mutant groups. The mRNA expression of two key genes associated with fatty acid oxidation (i.e., carnitine palmitoyltransferase 1A, encoded by *Cpt1a*; and peroxisome proliferator-activated receptor alpha, encoded by *Ppara*) was not significantly changed in either *ob/ob* or *db/db* mice, suggesting no changes in the fatty oxidation pathway (Fig. 2b).

To further investigate whether hepatic lipid steatosis was also associated with hepatic inflammation, we measured the mRNA expression of several markers associated with recruitment/infiltration of various types of the immune cell population (i.e., C-C motif chemokine ligand 2, encoded by *Ccl2*; adhesion G-protein-coupled receptor E1, encoded by *Adgre1*; integrin subunit alpha X, encoded by *Itgax*; cluster of differentiation 68, encoded by *Cd68*; and cluster of differentiation 163, encoded by *Cd163*). In the *ob/ob* mice, we observed a significant upregulation of the mRNA expression of *Ccl2* (a chemokine that regulates migration and infiltration of monocytes/macrophages), *Adgre1* (a marker reflecting the total number of mature macrophages), *Itgax* (a marker of dendritic cells), and *Cd68* (a marker of monocytes/macrophages), while a reduction of the expression of *Cd163* (a marker of anti-inflammatory monocyte/macrophages), barely failed to attain statistical significance (*P* = 0.060) in *ob/ob* mice compared to *db/db* and lean mice (Fig. 2c). We next confirmed an 84.1% increase of macrophage infiltration in the liver of *ob/ob* mice compared to *db/db* mice by performing a F4/80 immunostaining and counting crown-like structures (CLSs, i.e. macrophages surrounding dead or dying hepatocytes with large lipid droplets) on hepatic slices (Fig. 2d). Consistently with the higher immune cell recruitment, the mRNA expression of key receptors involved in the recognition of pathogen-associated molecules patterns of Gram-negative bacteria (i.e., cluster differentiation 14, encoded by *Cd14*; toll-like receptor 4, encoded by *Tlr4*; toll-like receptor 2, encoded by *Tlr2*; NLR family pyrin domain containing 3, encoded by *Nlrp3*), and of pro-inflammatory cytokines (i.e., tumor necrosis factor alpha, encoded by *Tnf*; interleukin 1 beta, encoded by *Il1b*) were significantly upregulated in *ob/ob* mice compared to *db/db* mice (Fig. 2e), while no changes in the mRNA expression of toll-like receptor 5 (encoded by *Tlr5*) were observed (Fig. 2e). These results suggest a severe liver inflammation associated with massive recruitment of immune cells in *ob/ob* mice. Given that chronic liver inflammation leads to fibrosis [35], we also investigated the expression of fibrosis-related genes (i.e., collagen type I alpha 1 chain, encoded by *Col1a1*; and transforming growth factor beta, encoded by *Tgfb1*). The expression of both genes was significantly increased in the *ob/ob* mice compared to the *db/db* mice (Fig. 2f). Altogether, these results highlight a different hepatic profile in terms of steatosis, inflammation, and fibrosis between *ob/ob* and *db/db* mice.

### Different bile acid metabolism and bile acid profile between *ob/ob* and *db/db* mice

Hepatic inflammation can be triggered by several stimuli. Gut-derived endotoxin such as lipopolysaccharides (LPS, components of Gram-negative bacteria outer membrane) can reach the liver via the portal circulation and promote the release of large amounts of proinflammatory mediators via its receptor, TLR4 [7]. Additionally, cholestasis, i.e., a decrease in bile flow due to impaired secretion by hepatocytes or to obstruction of bile flow through the bile ducts, can lead to accumulation of bile acids in the liver and thereby contribute to inflammation [36]. For this reason, we measured the serum LPS concentration and the BA content in the liver of both *ob/ob* and *db/db* mice, and their respective lean littermates. Strikingly, we found a significant 32.5% increase of serum LPS concentration in the *db/db* mice compared to the *ob/ob* mice (Fig. 3a), and consistent with our hypothesis, the amount of cholic acid (CA), a major primary free BA, was 94.5% significantly increased in the liver of *ob/ob* mice compared to the *db/db* mice. Conversely, there were no significant variations in the content of taurocholic acid (TCA), taurochenodeoxycholic acid (TCDCA), taurodeoxycholic acid (TDCA), tauroursodeoxycholic acid (TUDCA), tauro-alpha-beta muricholic acid (T(a+b) MCA) and tauro-omega muricholic acid (ToMCA) in the liver of both *ob/ob* and *db/db* mice (Fig. 3b).

Given that the BA profile is regulated by several mechanisms, we measured a large panel of markers associated with BA metabolism (i.e., synthesis, transport, and pool size) [37]. In the *ob/ob* mice, we observed a significant downregulation in the mRNA expression of markers involved in the classical (neutral) and the alternative (acidic) bile acid synthesis as well as in the CA production (i.e., cytochrome P450, family 8, subfamily B, member 1, encoded by *Cyp8b1*; and cytochrome P450, family 27, subfamily A, member 1, encoded by *Cyp27a1*), (Fig. 3c), while the mRNA expression of a rate-limiting enzyme of BA synthesis (i.e., cytochrome P450, family 7, subfamily A, member 1, encoded by *Cyp7a1*) tended to be decreased in *ob/ob* mice (Fig. 3c). Following BA synthesis, primary BAs are conjugated to taurine in mice by the enzymes bile acid CoA ligase (BAL) and bile acid CoA:amino acid N-acyltransferase (BAT) in order to increase their solubility for biliary secretion. Both enzymes are under the regulation of the hepatocyte nuclear factor 4 alpha (HNF4α) [38]. We observed in *ob/ob* mice a significant downregulation in the mRNA expression of *Slc27a5* (coding for BAL), and of *Hnf4a* (coding for HNF4α), while no changes in *Baat* (coding for BAT) occurred (Fig. 3c). These results suggest an impaired BA synthesis and conjugation in the *ob/ob* mice. We also measured several markers involved in either cholesterol, phospholipids transports, or BA reabsorption. We found that *Abcg5/8* (coding for cholesterol transporters ATP binding cassette, subfamily G, member 5, and 8) were significantly downregulated in the *ob/ob* mice compared to the *db/db* mice, whereas *Abcb4* mRNA (coding for the phospholipid transporter MDR2) was significantly increased in the *ob/ob* mice. The *Abcb11* mRNA (coding for bile salt export pump of hepatocytes BSEP) remained unaffected when comparing *ob/ob* and *db/db* (Fig. 3d), whereas the expression of *Slc51b* (coding for the transcellular transport of bile acids OSTβ), was significantly increased in *ob/ob* mice (Fig. 3d). The majority of the conjugated primary BAs are reabsorbed in the distal ileum and shuttled from the enterocytes into the portal circulation, where they are taken up by the hepatocytes and re-secreted into bile. In order to investigate the enterohepatic circulation, we measured the expression of several transporters implicated in this path. We found that the hepatic expression of *Slc10a1* (coding for the sodium (Na^+^) taurocholate cotransporting polypeptide NTCP)) and *Oatp1b2* (coding for the organic anion transporter OATP1B2) was significantly downregulated in the *ob/ob* mice compared to the *db/db* mice (Fig. 3e), whereas the ileal expression of *Slc10a2* (coding for the apical sodium-dependent bile salt transporter ASBT), *Fabp6* (coding for the bile acid-binding protein IBABP), and *Scl51b* were not significantly affected in either *ob/ob* or *db/db* mice (Fig. 3f). *Slc51a* mRNA (coding for the transcellular transport of bile acids OSTα) was the only marker to be slightly increased (*P* = 0.066) in the *db/db* mice compared to *ob/ob* mice (Fig. 3f).

Altogether, these results highlight an impaired BA metabolism associated with a different bile acid content between *ob/ob* and *db/db*. We hypothesized that not only LPS but also the hepatic BA accumulation may be the trigger of the changes observed above, thereby impairing the normal BA metabolism as well as the normal enterohepatic circulation of the BA.

### Different inflammatory profile in the subcutaneous adipose tissue between *ob/ob* and *db/db* mice

Body fat distribution and adipose tissue dysfunction are key factors involved in the development of obesity and its related metabolic disorders [39]. Because the metabolic, endocrine, and inflammatory profile of adipose tissue is depot dependent [40], we extensively characterized crucial markers related to the recruitment/infiltration of various types of immune cells, inflammation, and lipid metabolism, in two different and representative adipose tissue depots (SAT and VAT). Intriguingly, and in contrast to that observed in the liver, we found that the mRNA expression of *Ccl2*, *Adgre1*, and *Cd68*, was significantly increased in the SAT of *db/db* mice compared to the *ob/ob* mice, while no differences in the mRNA expression of *Itgax* (upregulated both in *ob/ob* and *db/db* mice) and *Cd163* were observed (Fig. 4a). The same tendencies for these markers were observed in the VAT of *db/db* mice (Additional file 3: Fig. S2a-b). To further confirm the increased macrophage infiltration into the SAT, immunohistochemical F4/80 staining showed that *db/db* mice presented a 34.5% increase in the number of CLSs compared to the *ob/ob* mice (Fig. 4b). CLSs formed by proinflammatory macrophages are found around large dying adipocytes during a state of obesity and have been associated with inflammation and insulin resistance both in mice and humans [41–44]. Along with the increased number of immune cells, the mRNA expression of *Il1b* and *Ifng* (coding for interferon gamma), two important proinflammatory cytokines, was significantly increased in the *db*/*db* mice compared to the *ob/ob* mice, while no significant changes in the expression of *Tlr4*, *Tlr2* (Fig. 4c) occurred. Interestingly, the mRNA expression of *Tlr5*, a key receptor involved in the recognition of pathogens-associated molecular patterns from Gram-positive bacteria (i.e., flagellin) was significantly increased in the *ob/ob* compared to the *db/db* mice (Fig. 4c). However, its increased expression was not associated with inflammation in the SAT of *ob/ob* mice. Additionally, the expression of *Ptgs2* (coding for prostaglandin-endoperoxidase synthase 2), a rate-limiting enzyme for prostaglandin production, which is implicated primarily in the regulation of inflammation in the white adipose tissue, was significantly increased in the *db/db* mice compared to the *ob/ob* mice (Fig. 4c). In the VAT, the expression of *Il6*, a major proinflammatory cytokine, was the only marker to be significantly increased in the *db/db* mice, while no significant differences were observed in the expression of other markers (i.e., *Tlr4*, *Tlr2*, *Tlr5*, *Il1b*) between *ob/ob* and *db/db* mice (Additional file 3: Fig. S2a-b). It is well established that proinflammatory cytokines play a crucial role in the regulation of adipogenesis, thereby influencing the formation of new adipocytes [45]. For that reason, we used quantitative PCR to determine the mRNA expression of key master regulators of the adipogenesis such as *Pparg* and *Cebpa* (coding for CCAAT enhancer-binding protein alpha), and fundamental markers involved in lipid synthesis (i.e., *Acaca*, *Fasn*)*.* We observed that *Cebpa* was significantly reduced in the *db/db* mice compared to the *ob/ob* mice, while the other markers tended to be downregulated to a greater extent in the *db/db* than in the *ob/ob* mice (Fig. 4d). No significant changes were observed for *Cpt1a* and *Ppara* mRNA expression between *ob/ob* and *db/db* mice, suggesting no changes in the lipid oxidation (Fig. 4d). These results mainly suggest an impaired adipocyte differentiation in the *db/db* mice.

### Different short-chain fatty acids and gut microbiota profile between *ob/ob* and *db/db* mice

Changes in gut bacteria-derived metabolites and gut microbiota composition could also participate in the different effects described above. SCFAs are the most abundant bacterial metabolites present in the gastrointestinal tract, which are involved in the regulation of several metabolic pathways [10]. In the present study, the amount of SCFAs was analyzed in the cecal content. Despite changes in the morphology of the cecum, there were no significant differences in the cecum weight, cecal content weight, and cecal tissue weight between *ob/ob* and *db/db* mice (Fig. 5a). On the other hand, we found that the amount of acetic acid, butyric acid (Fig. 5b), isobutyric acid, and hexanoic acid (Fig. 5c) was significantly decreased in the *db/db* mice compared to the *ob/ob* mice (36.4%, 36.9%, 40.7%, and 84%, respectively). No significant differences in the amount of propionic acid (Fig. 5b), 2-methylbutyric acid, valeric acid, and isovaleric acid between *ob/ob* and *db/db* mice were observed (Fig. 5c). Furthermore, when taking into consideration all the metabolic parameters, the principal component analysis (PCA) showed that the two control groups clustered together, while there is a clear separation between the two mutant groups (Fig. 5d), strongly emphasizing their metabolic diversity. PCA resulted in three principal components, explaining respectively 38%, 15%, and 7% of the total variance in the data set. The first principal component was correlated with overall weight-related metabolic parameters, explaining the difference between the control groups and experimental groups. For the second principal component (PC2), which explained the difference between the *ob/ob* and *db/db* experimental groups, the liver and SAT gene expressions had contrasting loadings. This indicates that the two mutant models can be differentiated based on their metabolic parameter profile and that inflammation of the liver (for *ob/ob*) and inflammation of SAT (for *db/db*) explains this differentiation. Moreover, cecal content of SCFAs had a positive loading for PC2, explaining its lower abundance in the *db/db* model.

**Fig. 6:**
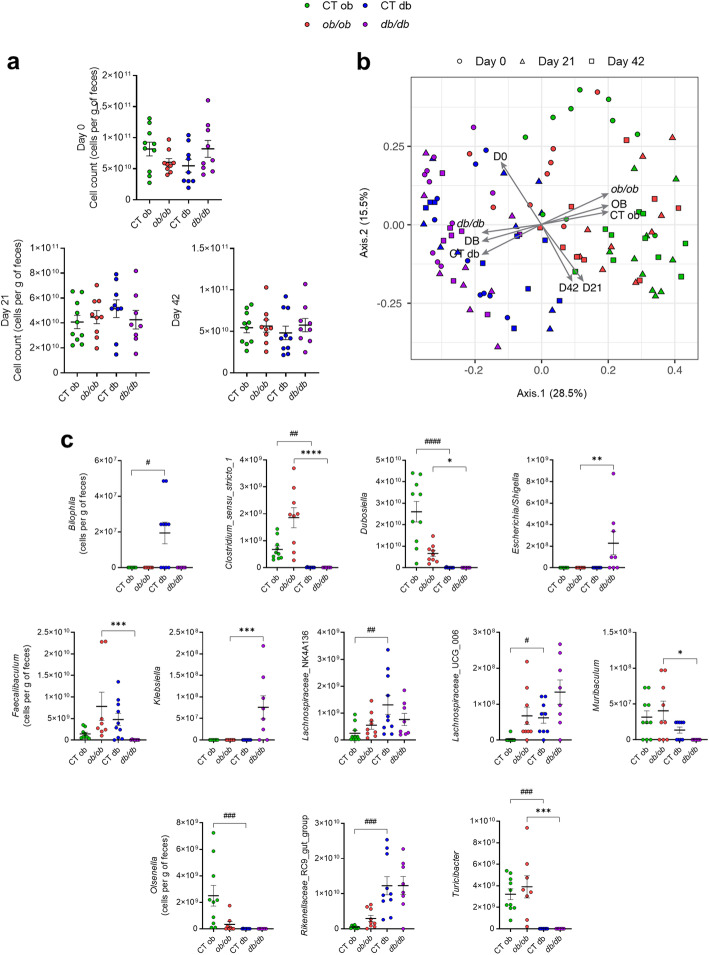
Similar fecal microbial load but different quantitative gut microbiota profiles among the four genotype groups. (**a**) Microbial load (cells/g of feces) at day 0, day 21, and day 42 measured by flow cytometry (n = 8–10). (**b**) Genus-level fecal microbiome community variation, represented by principal coordinates analysis (Bray-Curtis dissimilarity PCoA) (n = 112). Arrows correspond to a post hoc fit of the mouse groups on the PCoA. (**c**) Genera displaying significant quantitative abundance differences between mouse genotypes at day 42 (n = 7–10). Genera with a prevalence across samples lower than 15% were excluded. Data are presented as the mean ± s.e.m, ^#,^ *P< 0.05, ^##^*P* < 0.01, ^###,^ ***P < 0.001, ^####,^ ****P <0.0001. Green: CT ob lean mice, red: *ob/ob* mice, blue CT db lean mice, and violet: *db/db* mice. Data were analyzed by the Kruskal-Wallis test with Dunn’s multiple comparison test for (**a**) and (**c**)

Given that *ob/ob* and *db/db* were fed the same control diet for the full experiment, these results suggest that the different SCFA profiles are not diet-related but could reflect a different gut microbiota profile between *ob/ob* and *db/db*. To that end, we first determined the total microbial cell count in fecal samples collected on three different days (day 0, day 21, day 42) using flow cytometry. We found no difference in the feces total microbial density between *ob/ob* and *db/db* mice in the three different days as well as for the lean littermate groups (Fig. 6a). Second, we combined amplicon sequencing (16S rRNA gene) with experimentally measured microbial loads to obtain quantitative microbiota profiles for both *ob/ob* and *db/db* mice and their respective littermates using fresh feces collected during the same days as the microbial load. We also investigated microbiota alpha-diversity, and there was no significant difference in richness observed between days (Kruskal-Wallis *P* = 0.49) or mice groups (*P* = 0.12). Microbiota genus-level compositional variation, as visualized in a principal coordinates analysis (PCoA; Bray-Curtis dissimilarity; Fig. 6b), revealed a distinct clustering between the *ob/ob* and the *db/db* groups (permutational analysis of variance Adonis test; R^2^ = 0.248, *P* = 1e−05, N = 53) as well as between the two control groups (Adonis test; R^2^ = 0.261, *P* = 1e−05, N = 59) across sampling days. These four mice groups explained 29.5% of overall fecal microbiota variation, while sampling day added 7.1% explained variance within groups (Adonis test [groups + days]; *P* = 1e−05, N = 112). When looking at the gut microbiota composition, we observed specific taxa differences between mice groups. Despite a distinct gut microbiota composition between the mice groups already at day 0 (Adonis test; R^2^ = 0.354, *P* = 1e−05, N = 37), we identified several taxa that shift in abundance by day 42 in both *ob/ob* and *db/db* mice as well as between the two control groups (Fig. 6c). We found that the quantity of 19 genera was significantly (*Clostridium*_*sensu*_*stricto*_1, *Dubosiella*, *Escherichia*/*Shigella*, *Faecalibaculum*, *Klebsiella*, *Muribaculum,* and *Turicibacter*) (Fig. 6c and Additional file 4: Table S2), or tended (i.e., A2, *Bacteroides, Lachnospiraceae, Lachnoclostridium, Lactobacillus, Lactococcus, Lachnospiraceae_*FCS020, *Marvinbryantia*, *Ruminoclostridium*, *Ruminoclostridium* 5, *Shuttlerworthia*, and *Tyzzerella*) (Additional file 5: Fig. S3) to be affected by either the *ob/ob* or the *db/db* genotype or by both. Surprisingly, we also observed that the quantity of 11 other genera was significantly different between the two control groups (*Bilophila*, *Clostridium*_*sensu*_*stricto*_1, *Dubosiella*, *Lachnospiraceae*_NK4A136_group, *Lachnospiraceae*_UCG.006, *Olsenella*, *Rikenellaceae*_RC9_gut group, *Turicibacter*) (Fig. 6c and Additional file 4: Table S2), or tended to be (i.e., *Akkermansia muciniphila*, *Parabacteroides*, and *Ruminococcaceae*_UCG_014) (Additional file 5: Fig S3). Altogether, these results highlight a different gut microbiota profile and composition not only between the two mutant mice, but also between their respective controls, although displaying the same lean and non-diabetic phenotype. Given the important role in the cross-talk between gut microbes and host, we then sought to correlate the bacterial genera with various metabolic parameters (Additional file 6: Table S3). In particular, we identified *Akkermansia muciniphila* and *Shuttleworthia* as the two genera to be the most negatively (*A. muciniphila*) and positively (*Shuttleworthia*) correlated with body weight, glucose profile, lipid metabolism, bile acid metabolism, and liver and adipose tissue inflammation.

## Discussion

*Ob/ob* and *db/db* mice are widely used as animal models to investigate the pathogenesis of metabolic diseases such as obesity and T2D. However, although both animal models rely on the disruption of the leptin signaling pathway by targeting the ligand (*ob/ob*) or the receptor (*db/db*), and both models are characterized by hyperphagia, massive obesity, and fat mass gain, they are discrepant for glucose metabolism. So far, the origin of these phenotypical differences is unknown. To this aim, in the present study, we extensively characterized these mice. Although both *ob/ob* and *db/db* mice had equivalent evolutions in terms of body weight and fat mass gain, we found they had quite distinctive metabolic features, thereby decoupling the observed metabolic features from the obese phenotype. Besides being diabetic, *db/db* mice had higher food intake, and therefore a lower feeding efficiency, than *ob/ob* mice. This is likely explained by several mechanisms, such as the loss of glucose in the urine during polyuria, the higher energy excretion in the feces, and the lower body temperature. In agreement with our study, Giesbertz et al. have previously shown that despite the same body weight, *ob/ob* and *db/db* mice had a different metabolite profiling in plasma and tissues [18]. However, the authors did not further investigate the origins of these differences. In the present study, we discovered that several important features such as the inflammatory tone in different tissues, the gut microbiota composition, bacterial components (i.e., LPS), bacteria-derived metabolites, as well as different bioactive lipids (i.e., bile acids) allowed discriminating the *db/db* from the *ob/ob* mice. Therefore, our data further explain the difference between the two phenotypes and have led to the identification of novel markers.

*Ob/ob* mice develop an altered hepatic lipid metabolism, with a higher hepatic steatosis and inflammatory tone characterized by a marked increase in immune cell infiltration. We have explored several mechanisms that could account for this phenotype.

We and others have previously demonstrated in *ob/ob* mice that the inflammatory phenotype observed in the adipose tissue as well as liver dysfunction is closely linked to the gut microbiota, since its depletion using antibiotics lowers endotoxemia-induced inflammation and related metabolic disorders [46, 47]. A previous study in *db/db* mice fed with a standard chow diet also showed that the leakage of gut microbiota-derived LPS into the portal blood is a well-established mechanism of metabolic endotoxemia that promotes liver damage [16]. These findings were in contrast with our study, in which *db/db* mice were protected from liver damage. Differences in experimental procedures (i.e., different diet composition, ages, duration of the experiment) may explain the discrepancies between the studies. However, bacterial components such as LPS are not the only cause of liver damage. Other factors, such as the BA, are also involved in the regulation of innate immunity and liver function [48], and cholestasis, which is an impaired bile flow leading to accumulation of bile acids in the liver, can also promote liver inflammation. In our study, we observed that the hepatic level of cholesterol, the precursor for BA synthesis, was significantly increased in *ob/ob* mice. Strikingly, cholic acid (CA) levels were 94.5% higher in *ob/ob* than in *db/db* mice, whereas the other BA were comparable between both genotypes. As a matter of fact, the expression of main enzymes involved in the classical pathway of the BA synthesis (*Cyp7a1*, *Cyp8b1*, *Cyp27a1*) was downregulated in *ob/ob* mice and all other markers were pointing towards a lower BA conjugation, higher BA excretion, and lower BA reabsorption. The downregulation of those markers could be interpreted as a protecting mechanism of the liver from the toxic effect of bile acid accumulation. Additionally, we observed that the hepatic *Slc51b* expression, a basolateral organic solute transporter that mediates bile acid efflux, was significantly increased in *ob/ob* mice. Given the significant role exerted by the enterohepatic circulation in the regulation of the BA synthesis [49], we found that the expression of transporters in the ileum regulating the reabsorption of bile acids (*Slc10a2*, *Fabp6*, *Slc51a*, *Slc51b*) was unchanged in both mutant groups. Altogether, these data are in accordance with human and animal studies showing that during cholestasis, an alteration of the bile acid transporters occurs and is characterized by a downregulation of the uptake systems (NTCP, and OTAPs) and upregulation of basolateral bile acid export systems (OSTβ) (reviewed in [50]). Bile acid signaling in the liver and in the intestine is now considered a potential target for the treatment of obesity and non-alcoholic fatty liver disease (NAFLD) [51]. The role of bile acid in inducing liver injury is mainly evidenced by the use of bile acid sequestrants, whose use reversed liver injury and prevented the progression of steatosis, inflammation, and fibrosis in mice fed a Western diet-induced non-alcoholic steatohepatitis (NASH) mouse model [52]. Furthermore, given the bidirectional link between bile acids and gut microbiota composition, we cannot exclude that a disruption of the bacterial gut community may affect bile acid synthesis in the liver. A previous study in mice has shown that the gut microbiota not only regulates secondary bile acid metabolism but also inhibits bile acid synthesis in the liver by alleviating farnesoid X receptor (FXR) inhibition in the ileum [53]. Hence, we may not exclude the role of the gut microbiota as an explanation of our results as further discussed below.

Unlike the relatively low inflammation observed in the liver of *db/db* mice compared to *ob/ob* mice, we found that *db/db* mice had a higher inflammatory tone in the adipose tissue than *ob/ob* mice. Several potential mechanisms have emerged as the main trigger in the onset of adipose tissue inflammation, including gut-derived substances, dietary component, metabolites, and adipocyte death (reviewed in [54]). Despite no change in the expression of the TLRs (i.e., TLR4, and TLR2), we may speculate that the downregulation in the expression of fundamental markers associated with adipocyte differentiation (*Pparg*, *Cebpa*)*,* may explain adipocyte death, recruitment of immune cells, and production of proinflammatory cytokines, thereby triggering adipose tissue inflammation and insulin resistance in *db/db* mice. We have previously shown *in vivo* and *in vitro* that LPS acts as a master switch to control adipose tissue metabolism and its plasticity during obesity [55]. However, SCFAs, whose concentrations were reduced in the cecal content of *db/db* mice, could also be involved. Several studies *in vitro* and *in vivo* have shown their effects on immunity, inflammation, and adipose tissue expansion [56–58]. Here, we found that the concentration of SCFAs in the cecal content was not significantly increased in *ob/ob*. This observation is not in line with a previous study in *ob/ob* mice having shown that changes in gut microbiota composition were associated with an increased concentration of SCFAs (i.e., butyrate, and acetate) in the cecal content and less energy content in the stool of the mutant mice [59]. Contrary to these findings, we found a higher energy excretion in the feces of both *ob/ob* and *db/db* mice compared to their respective control groups. Therefore, in our context, it is unlikely that the SCFAs account for the differences in obese phenotypes. Intriguingly, we observed a significant increase in the amount of hexanoic acid in the cecal content of the *ob/ob* mice compared to the *db/db* mice. So far, there are no studies describing its role in the onset of obesity development as well as in the regulation of liver and adipose tissue function and metabolism, and further studies are needed to confirm its function. Certain SCFAs, such as acetate, have been shown to modulate appetite in mice [60]. This could explain the higher food intake observed in *db/db* mice. Given the important role of the gut microbiota in all the metabolic functions mentioned above, we decided to study the overall microbial community in depth using a recently developed method combining amplicon sequencing and flow cytometry: quantitative microbiome profiling (QMP). Microbial load, defined as the total number of bacteria in a given quantity of sample, was proposed as a main driver of microbiota alteration as shown in a cohort of patients with inflammatory bowel disease [31]. Here, we did not observe significant differences in the microbial load between *ob/ob* and *db/db* mice over the three different time points, thereby excluding this factor as a major driver of the phenotype. By doing QMP, we demonstrate that some genera are more present in the *ob/ob* mice compared to the *db/db* mice, and vice versa, and we discovered new genera that may be implicated in the onset of these pathological conditions.

In the present study, we identified that the quantity of *Clostriudium_sensu_stricto*_1, *Dubosiella*, *Faecalibaculum*, *Turicibacter* (Gram-positive bacteria of the phylum Firmicutes), and *Muribaculum* (Gram-negative bacteria of the phylum Bacteroidetes) was significantly higher in *ob/ob* mice when compared to the *db/db* mice. A recent human study has shown that *Clostridium_sensu_stricto*_1 is positively correlated with indicators of body weight and serum lipids [61], while *Faecalibaculum* and *Muribaculum* are two recently identified bacteria that have been isolated from the feces and the intestine of murine models respectively [62, 63]. So far, there are no studies describing the relationship between *Faecalibaculum* in the context of obesity and related metabolic disorders, while there is one recent study showing a higher proportion of OTUs most closely related to *Muribaculum* species in BA fed mice [64], and another recent one showing a lower proportion of *Muribaculum intestinalis* in mice fed with high-fat diet, high-glucose diet, and high-fructose diet [65]. Consistent with our observations, data from other studies observed a higher abundance of *Lactobacillus* in *ob/ob* mice [66]. The increase in *Lactobacillus* was unexpected as this genus is usually considered a “beneficial bacterium.” However, several studies have already linked *Lactobacillus* spp*.* with obesity [67–69]. It cannot be excluded that differences in the abundance of this bacterial taxa may also reflect the distinct food intake and energy excreted in the feces observed between *ob/ob* and *db/db* mice. Moreover, we found positive correlations between *Lactobacillus* and the hepatic lipid content, bile acid metabolism, and inflammation markers, thereby suggesting that the role of *Lactobacillus* spp. needs further investigation in studies designed specifically for this purpose. Conversely to the *ob/ob*, in the *db/db* mice, we identified a higher quantity of certain Gram-negative bacteria such as *Bacteroides* (member of the phylum Bacteroidetes*)*, *Escherichia/Shigella*, *Klebsiella* (member of the phylum Proteobacteria), *Lachnospiraceae* (member of the phylum Firmicutes), and Gram-positive bacteria such as *Lactococcus*. A recent study in obese individuals with and without T2D showed that the participants with T2D, compared with participants in the obese non-diabetic group, displayed different microbial signatures with higher Proteobacteria members (that is, *Escherichia* and *Shigella*) in the plasma and mesenteric adipose tissue. This observation also corroborates data showing higher abundance of *Escherichia* and *Shigella* in the feces of dysglycemic individuals compared with normoglycemic individuals [70]. Other recent human studies highlighted the presence of bacteria and bacterial DNA, mainly from Proteobacteria and Firmicutes, in several adipose tissues in obesity and T2D, thereby suggesting a critical role of bacteria in promoting and sustaining local adipose tissue subclinical inflammation and therefore affecting the different metabolic disorders linked to obesity [70, 71]. *Klebsiella*, another member of the Proteobacteria phylum, was also found to be enriched in obese children [72], and members of the *Lachnospiraceae* family have also been associated with T2D [73]. Along with our previous studies, we observed a lower quantity of *Akkermansia muciniphila* in *ob/ob* and even lower in *db/db* mice. This observation has also been confirmed in humans [74, 75]. Our group was the first to describe the ability of this bacterium to delay development of diet-induced obesity and insulin resistance in mice, namely via the modulation of the energy homeostasis and restoration of the gut barrier function [75]. More recently, in humans, we confirmed in a placebo-controlled study in overweight/obese insulin-resistant volunteers that supplementation with *A. muciniphila* could prevent the worsening of several metabolic parameters [76]. In addition to the different gut microbiota profiles between *ob/ob* and *db/db*, we also identified genera that differed between the two control groups. For example, a higher quantity of *A. muciniphila* in CT db mice, and higher quantity of *Dubosiella* and *Olsenella* in CT ob mice, among others.

*Dubosiella* has been recently isolated from the murine intestine and associated with protection from adiposity in mice [77]. Studies in both mice and humans have also described the association between increased physical activity and microbiome changes as well as SCFAs production [78], thus we may not rule out that the distinct microbiota profile between *ob/ob* and *db/db* mice and their lean counterparts may reflect a different locomotor activity that occurred over the duration of the experiment.

As shown in Fig. 6b and Fig. 5d despite a different microbiota composition, the two control groups clustered together when taking into consideration all the metabolic parameters, suggesting that the increase in certain beneficial bacteria plays an important role in the modulation of the metabolic function. Taking this together, we propose that the divergent shifts in gut microbial community contribute to the development of the two complex phenotypes, although further studies are needed to determine whether the associated microbial taxa have a causal effect on body weight, glucose profile, and inflammation. However, the reason for changes in the gut microbiota still remains unclear, despite unchanged genetic background and diet. Furthermore, the difference in the microbiota composition and bile acid profile are likely contributing to the different hepatic phenotypes observed between mice. We may not rule out that divergences in food intake and immune system activation could also have contributed to shape the gut microbiota composition. We also acknowledge that having used only male mice is a limitation of the present study. Indeed, the use of mice of both sexes would have provided additional metabolic information and further elucidate gender-related dissimilarities in the overall gut microbiota composition of genetically obese and diabetic mice.

## Conclusion

**Fig. 7:**
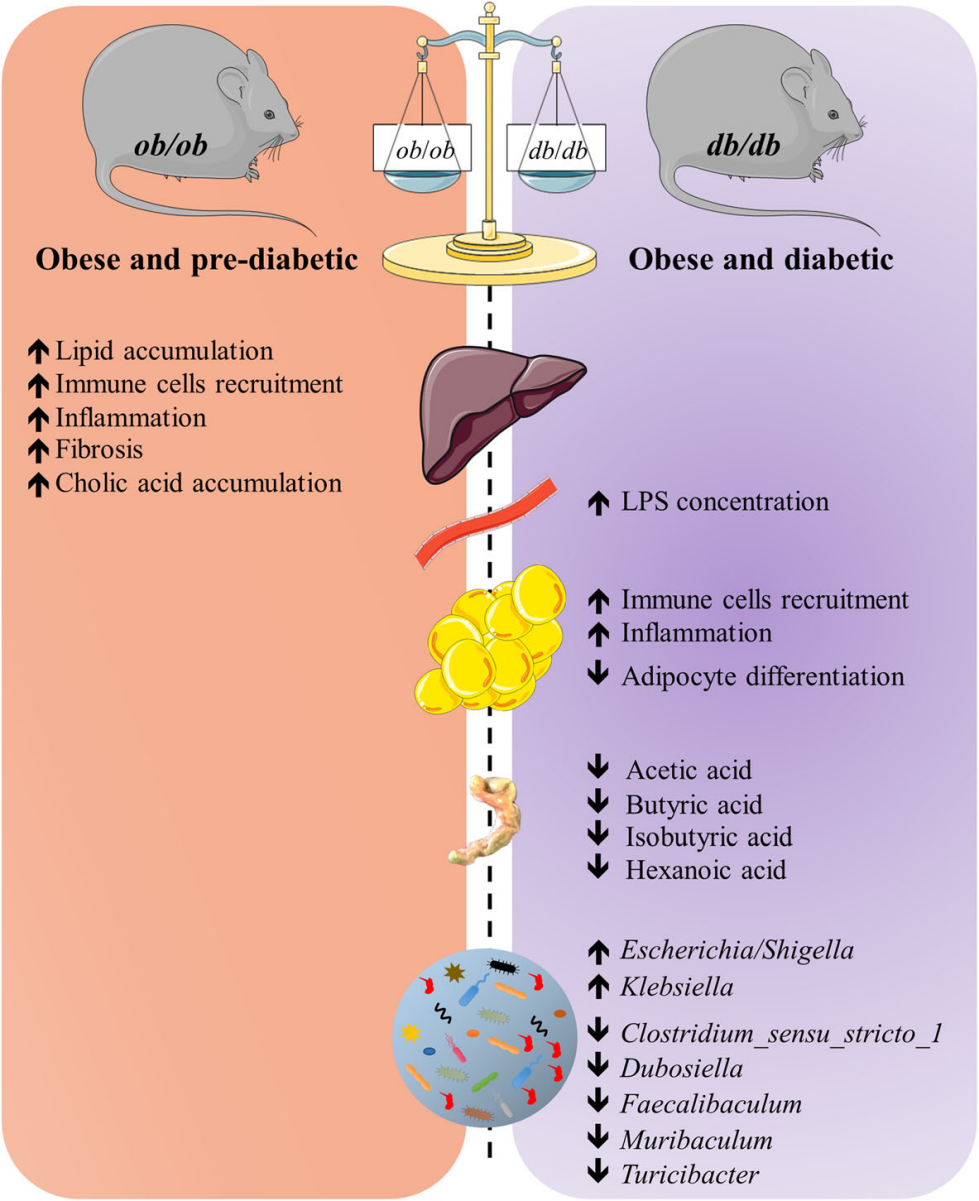
Graphical abstract. This figure summarizes the major differences observed between the two different models. Each specificity related to the organ of body fluid are depicted by a pictogram of the organ

Our results support that the unique metabolic features differentiating *ob/ob* and *db/db* mice are explained in part by severe differences in their gut microbiota compositions, gut bacterial components like the LPS, and gut-derived metabolites such as SCFAs, as well as in their bile acid profiles (Fig. 7). We also described a different inflammatory tone at two different biological sites, with the liver being more affected in *ob/ob* mice and the adipose tissue in *db/db* mice, thereby emphasizing that the development of obesity and diabetes is more organ-dysfunction (i.e., liver and adipose tissue) related. These findings further underscore the differences between the two mutant strains and emphasize that these are not interchangeable experimental models (Fig. 7). By discovering their specificities, connecting important biological markers, and identifying new bacteria, we open innovative opportunities for functional studies in the context of obesity and related metabolic disorders such as diabetes, liver injury, and adipose tissue inflammation.

## Supplementary Information

**Additional file 1: Table S1.** RT-qPCR primer sequences for the targeted mouse genes.

**Additional file 2: Fig. S1.** Different food intake and water intake profile, body temperature, feces production and energy excreted by feces in *ob/ob* and *db/db* mice. (**a**) Food intake evolution (g/mouse/day) measured for the entire experiment (n = 4-5). (**b**) Water intake evolution (mL/mouse/day) measured for the entire experiment (n = 4-5). (**c**) Body temperature (°C) (n = 9-10). (**d**) Feces produced per day (mg/mouse) (n = 4-5). (**e**) Caloric content (cal/g of feces) in 24h feces collected (n = 4-5). (**f**) Energy excreted by feces (cal/g of feces/24h) (n = 4-5). Green: CT ob lean mice, red: *ob/ob* mice, blue CT db lean mice, and violet: *db/db* mice. Data are presented as the mean ± s.e.m, *P < 0.05, **P < 0.01, ***P < 0.001, ****P < 0.0001. Data were analyzed by one-way ANOVA followed by Tukey’s post hoc test.

**Additional file 3: Fig. S2.** Similar visceral adipose tissue features between *ob/ob* and *db/db* mice. (**a**) mRNA expression of VAT immune cells markers measured by RT-qPCR. (**b**) mRNA expression of VAT receptors and inflammatory cytokines markers measured by RT-qPCR. Green: CT ob lean mice, red: *ob/ob* mice, blue CT db lean mice, and violet: *db/db* mice. Data are presented as the mean ± s.e.m., **P < 0.01 (n = 8-10). For the mRNA expression, relative units were calculated versus the mean of the CT ob mice values set at 1. Data were analyzed by one-way ANOVA followed by Tukey’s post hoc test.

**Additional file 4: Table S2.** Genera displaying significant quantitative abundance differences between mouse genotypes at day 42 (n = 37, Kruskal-Wallis and post-hoc Dunn test). Genera with a prevalence across samples lower than 15% were excluded. Multiple testing correction was performed (BH method).

**Additional file 5: Fig. S3.** Different quantitative gut microbiota profiles among the four genotype groups. Green: CT ob lean mice, red: *ob/ob* mice, blue CT db lean mice, and violet: *db/db* mice. Data are presented as the mean ± s.e.m, (n = 7–10). Genera with a prevalence across samples lower than 15% were excluded. Data were analyzed by Kruskal-Wallis test with Dunn’s multiple comparison test.

**Additional file 6: Table S3.** Taxa-metabolic parameters associations. Spearman correlation between bacterial genera and selected metabolic parameters. Genera whose prevalence was less than 15% of the samples were excluded. Multiple testing correction was performed (Benjamini-Hochberg method).

**Additional file 7: Table S4.** Processed quantitative microbiota matrix of day 0, 21, 42.

## Data Availability

All data generated or analyzed during this study are included in this published article and its supplementary information files. The raw amplicon sequencing data analyzed in this study have been deposited in the European Nucleotide Archive (ENA) at EMBL-EBI under accession number PRJEB44809 (https://www.ebi.ac.uk/ena/browser/view/PRJEB44809). The processed quantitative microbiota matrix is provided as Additional file 7: Table S4.

## Abbreviations

T2D: Type 2 diabetes
BA: Bile acids
SCFAs: Short-chain fatty acids
QMP: Quantitative microbial profiling
SAT: Subcutaneous adipose tissue
VAT: Visceral adipose tissue
CLSs: Crown-like structures
OGTT: Oral glucose tolerance test
TLRs: Toll-like receptors
LPS: Lipopolysaccharides
CA: Cholic Acid

## References

[1] Collaboration, N.C.D.R.F (2016). Trends in adult body-mass index in 200 countries from 1975 to 2014: a pooled analysis of 1698 population-based measurement studies with 19.2 million participants. Lancet.

[2] Grieve E, Fenwick E, Yang HC, Lean M (2013). The disproportionate economic burden associated with severe and complicated obesity: a systematic review. Obes Rev.

[3] O'Neill S, O'Driscoll L (2015). Metabolic syndrome: a closer look at the growing epidemic and its associated pathologies. Obes Rev.

[4] Bäckhed F (2004). The gut microbiota as an environmental factor that regulates fat storage. Proc Natl Acad Sci USA.

[5] Backhed F (2007). Mechanisms underlying the resistance to diet-induced obesity in germ-free mice. Proc Natl Acad Sci U S A.

[6] Bluher M (2019). Obesity: global epidemiology and pathogenesis. Nat Rev Endocrinol.

[7] Cani PD, Amar J, Iglesias MA, Poggi M, Knauf C, Bastelica D, Neyrinck AM, Fava F, Tuohy KM, Chabo C, Waget A, Delmee E, Cousin B, Sulpice T, Chamontin B, Ferrieres J, Tanti JF, Gibson GR, Casteilla L, Delzenne NM, Alessi MC, Burcelin R (2007). Metabolic endotoxemia initiates obesity and insulin resistance. Diabetes.

[8] Cani PD, Osto M, Geurts L, Everard A (2012). Involvement of gut microbiota in the development of low-grade inflammation and type 2 diabetes associated with obesity. Gut Microbes.

[9] Cani PD, van Hul M, Lefort C, Depommier C, Rastelli M, Everard A (2019). Microbial regulation of organismal energy homeostasis. Nat Metab.

[10] Canfora EE, Jocken JW, Blaak EE (2015). Short-chain fatty acids in control of body weight and insulin sensitivity. Nat Rev Endocrinol.

[11] Wahlstrom A (2016). Intestinal Crosstalk between Bile Acids and Microbiota and Its Impact on Host Metabolism. Cell Metab.

[12] Suriano F, Van Hul M, Cani PD (2020). Gut microbiota and regulation of myokine-adipokine function. Curr Opin Pharmacol.

[13] Friedman JM, Halaas JL (1998). Leptin and the regulation of body weight in mammals. Nature.

[14] Wang B, Chandrasekera PC, Pippin JJ (2014). Leptin- and leptin receptor-deficient rodent models: relevance for human type 2 diabetes. Curr Diabetes Rev.

[15] Wauman J, Zabeau L, Tavernier J (2017). The Leptin Receptor Complex: Heavier Than Expected?. Front Endocrinol (Lausanne).

[16] Brun P, Castagliuolo I, Leo VD, Buda A, Pinzani M, Palù G, Martines D (2007). Increased intestinal permeability in obese mice: new evidence in the pathogenesis of nonalcoholic steatohepatitis. Am J Physiol Gastrointest Liver Physiol.

[17] Everard A, Lazarevic V, Derrien M, Girard M, Muccioli GG, Neyrinck AM, Possemiers S, van Holle A, François P, de Vos WM, Delzenne NM, Schrenzel J, Cani PD (2011). Responses of gut microbiota and glucose and lipid metabolism to prebiotics in genetic obese and diet-induced leptin-resistant mice. Diabetes.

[18] Giesbertz P, Padberg I, Rein D, Ecker J, Höfle AS, Spanier B, Daniel H (2015). Metabolite profiling in plasma and tissues of ob/ob and db/db mice identifies novel markers of obesity and type 2 diabetes. Diabetologia.

[19] Geurts L (2011). Altered gut microbiota and endocannabinoid system tone in obese and diabetic leptin-resistant mice: impact on apelin regulation in adipose tissue. Front Microbiol.

[20] Coleman DL (1978). Obese and diabetes: two mutant genes causing diabetes-obesity syndromes in mice. Diabetologia.

[21] Coleman DL, Hummel KP (1973). The influence of genetic background on the expression of the obese (Ob) gene in the mouse. Diabetologia.

[22] Yang M (2019). Gut Microbiota Composition and Structure of the Ob/Ob and Db/Db Mice. Int J Endocrinol.

[23] Folch J, Lees M, Sloane Stanley GH (1957). A simple method for the isolation and purification of total lipides from animal tissues. J Biol Chem.

[24] Everard A, Plovier H, Rastelli M, van Hul M, de Wouters d’Oplinter A, Geurts L, Druart C, Robine S, Delzenne NM, Muccioli GG, de Vos WM, Luquet S, Flamand N, di Marzo V, Cani PD (2019). Intestinal epithelial N-acylphosphatidylethanolamine phospholipase D links dietary fat to metabolic adaptations in obesity and steatosis. Nat Commun.

[25] Lefort C, et al. Hepatic NAPE-PLD Is a Key Regulator of Liver Lipid Metabolism. Cells. 2020;9(5):1247. 10.3390/cells9051247. 10.3390/cells9051247 PMC7291298 32443626

[26] Prest EI, Hammes F, Kötzsch S, van Loosdrecht MCM, Vrouwenvelder JS (2013). Monitoring microbiological changes in drinking water systems using a fast and reproducible flow cytometric method. Water Res.

[27] Vieira-Silva S, Sabino J, Valles-Colomer M, Falony G, Kathagen G, Caenepeel C, Cleynen I, van der Merwe S, Vermeire S, Raes J (2019). Quantitative microbiome profiling disentangles inflammation- and bile duct obstruction-associated microbiota alterations across PSC/IBD diagnoses. Nat Microbiol.

[28] Hildebrand F, Tadeo R, Voigt A, Bork P, Raes J (2014). LotuS: an efficient and user-friendly OTU processing pipeline. Microbiome.

[29] Callahan BJ, McMurdie PJ, Rosen MJ, Han AW, Johnson AJA, Holmes SP (2016). DADA2: High-resolution sample inference from Illumina amplicon data. Nat Methods.

[30] Quast C, Pruesse E, Yilmaz P, Gerken J, Schweer T, Yarza P, Peplies J, Glöckner FO (2013). The SILVA ribosomal RNA gene database project: improved data processing and web-based tools. Nucleic Acids Res.

[31] Vandeputte D, Kathagen G, D’hoe K, Vieira-Silva S, Valles-Colomer M, Sabino J, Wang J, Tito RY, de Commer L, Darzi Y, Vermeire S, Falony G, Raes J (2017). Quantitative microbiome profiling links gut community variation to microbial load. Nature.

[32] Stoddard SF, Smith BJ, Hein R, Roller BRK, Schmidt TM (2015). rrnDB: improved tools for interpreting rRNA gene abundance in bacteria and archaea and a new foundation for future development. Nucleic Acids Res.

[33] Revelle W (2020). Psych: Procedures for Psychological, Psychometric, and Personality Research. R package version 2.0.12.

[34] Oksanen J (2020). Vegan: Community Ecology Package. R package version 2.5-7.

[35] Koyama Y, Brenner DA (2017). Liver inflammation and fibrosis. J Clin Invest.

[36] Fickert P, Wagner M (2017). Biliary bile acids in hepatobiliary injury - What is the link?. J Hepatol.

[37] Sarenac TM, Mikov M (2018). Bile Acid Synthesis: From Nature to the Chemical Modification and Synthesis and Their Applications as Drugs and Nutrients. Front Pharmacol.

[38] Inoue Y, Yu AM, Inoue J, Gonzalez FJ (2004). Hepatocyte nuclear factor 4alpha is a central regulator of bile acid conjugation. J Biol Chem.

[39] Longo M, et al. Adipose Tissue Dysfunction as Determinant of Obesity-Associated Metabolic Complications. Int J Mol Sci. 2019;20(9):2358. 10.3390/ijms20092358. 10.3390/ijms20092358 PMC6539070 31085992

[40] Chait A, den Hartigh LJ (2020). Adipose Tissue Distribution, Inflammation and Its Metabolic Consequences, Including Diabetes and Cardiovascular Disease. Front Cardiovasc Med.

[41] Lumeng CN, DelProposto JB, Westcott DJ, Saltiel AR (2008). Phenotypic switching of adipose tissue macrophages with obesity is generated by spatiotemporal differences in macrophage subtypes. Diabetes.

[42] Strissel KJ, Stancheva Z, Miyoshi H, Perfield JW, DeFuria J, Jick Z, Greenberg AS, Obin MS (2007). Adipocyte death, adipose tissue remodeling, and obesity complications. Diabetes.

[43] Cinti S, Mitchell G, Barbatelli G, Murano I, Ceresi E, Faloia E, Wang S, Fortier M, Greenberg AS, Obin MS (2005). Adipocyte death defines macrophage localization and function in adipose tissue of obese mice and humans. J Lipid Res.

[44] Apovian CM, Bigornia S, Mott M, Meyers MR, Ulloor J, Gagua M, McDonnell M, Hess D, Joseph L, Gokce N (2008). Adipose macrophage infiltration is associated with insulin resistance and vascular endothelial dysfunction in obese subjects. Arterioscler Thromb Vasc Biol.

[45] Jiang N, Li Y, Shu T, Wang J (2019). Cytokines and inflammation in adipogenesis: an updated review. Front Med.

[46] Cani PD, Bibiloni R, Knauf C, Waget A, Neyrinck AM, Delzenne NM, Burcelin R (2008). Changes in gut microbiota control metabolic endotoxemia-induced inflammation in high-fat diet-induced obesity and diabetes in mice. Diabetes.

[47] Membrez M, Blancher F, Jaquet M, Bibiloni R, Cani PD, Burcelin RG, Corthesy I, Macé K, Chou CJ (2008). Gut microbiota modulation with norfloxacin and ampicillin enhances glucose tolerance in mice. FASEB J.

[48] Fiorucci S, Biagioli M, Zampella A, Distrutti E (2018). Bile Acids Activated Receptors Regulate Innate Immunity. Front Immunol.

[49] Chiang JYL (2017). Bile acid metabolism and signaling in liver disease and therapy. Liver Res.

[50] Halilbasic E, Claudel T, Trauner M (2013). Bile acid transporters and regulatory nuclear receptors in the liver and beyond. J Hepatol.

[51] Arab JP, Karpen SJ, Dawson PA, Arrese M, Trauner M (2017). Bile acids and nonalcoholic fatty liver disease: Molecular insights and therapeutic perspectives. Hepatology.

[52] Takahashi S, Luo Y, Ranjit S, Xie C, Libby AE, Orlicky DJ, Dvornikov A, Wang XX, Myakala K, Jones BA, Bhasin K, Wang D, McManaman JL, Krausz KW, Gratton E, Ir D, Robertson CE, Frank DN, Gonzalez FJ, Levi M (2020). Bile acid sequestration reverses liver injury and prevents progression of nonalcoholic steatohepatitis in Western diet-fed mice. J Biol Chem.

[53] Sayin SI, Wahlström A, Felin J, Jäntti S, Marschall HU, Bamberg K, Angelin B, Hyötyläinen T, Orešič M, Bäckhed F (2013). Gut microbiota regulates bile acid metabolism by reducing the levels of tauro-beta-muricholic acid, a naturally occurring FXR antagonist. Cell Metab.

[54] Reilly SM, Saltiel AR (2017). Adapting to obesity with adipose tissue inflammation. Nat Rev Endocrinol.

[55] Muccioli GG, Naslain D, Bäckhed F, Reigstad CS, Lambert DM, Delzenne NM, Cani PD (2010). The endocannabinoid system links gut microbiota to adipogenesis. Mol Syst Biol.

[56] Hernandez MAG, et al. The Short-Chain Fatty Acid Acetate in Body Weight Control and Insulin Sensitivity. Nutrients. 2019;11(8):1943. 10.3390/nu11081943. 10.3390/nu11081943 PMC6723943 31426593

[57] Wang X, He G, Peng Y, Zhong W, Wang Y, Zhang B (2015). Sodium butyrate alleviates adipocyte inflammation by inhibiting NLRP3 pathway. Sci Rep.

[58] Al-Lahham S, Rezaee F (2019). Propionic acid counteracts the inflammation of human subcutaneous adipose tissue: a new avenue for drug development. Daru.

[59] Turnbaugh PJ, Ley RE, Mahowald MA, Magrini V, Mardis ER, Gordon JI (2006). An obesity-associated gut microbiome with increased capacity for energy harvest. Nature.

[60] Frost G, Sleeth ML, Sahuri-Arisoylu M, Lizarbe B, Cerdan S, Brody L, Anastasovska J, Ghourab S, Hankir M, Zhang S, Carling D, Swann JR, Gibson G, Viardot A, Morrison D, Louise Thomas E, Bell JD (2014). The short-chain fatty acid acetate reduces appetite via a central homeostatic mechanism. Nat Commun.

[61] Zeng Q, Li D, He Y, Li Y, Yang Z, Zhao X, Liu Y, Wang Y, Sun J, Feng X, Wang F, Chen J, Zheng Y, Yang Y, Sun X, Xu X, Wang D, Kenney T, Jiang Y, Gu H, Li Y, Zhou K, Li S, Dai W (2019). Discrepant gut microbiota markers for the classification of obesity-related metabolic abnormalities. Sci Rep.

[62] Lim S, Chang DH, Ahn S, Kim BC (2016). Whole genome sequencing of “Faecalibaculum rodentium” ALO17, isolated from C57BL/6J laboratory mouse feces. Gut Pathog.

[63] Lagkouvardos I, Pukall R, Abt B, Foesel BU, Meier-Kolthoff JP, Kumar N, Bresciani A, Martínez I, Just S, Ziegler C, Brugiroux S, Garzetti D, Wenning M, Bui TPN, Wang J, Hugenholtz F, Plugge CM, Peterson DA, Hornef MW, Baines JF, Smidt H, Walter J, Kristiansen K, Nielsen HB, Haller D, Overmann J, Stecher B, Clavel T (2016). The Mouse Intestinal Bacterial Collection (miBC) provides host-specific insight into cultured diversity and functional potential of the gut microbiota. Nat Microbiol.

[64] Just S, Mondot S, Ecker J, Wegner K, Rath E, Gau L, Streidl T, Hery-Arnaud G, Schmidt S, Lesker TR, Bieth V, Dunkel A, Strowig T, Hofmann T, Haller D, Liebisch G, Gérard P, Rohn S, Lepage P, Clavel T (2018). The gut microbiota drives the impact of bile acids and fat source in diet on mouse metabolism. Microbiome.

[65] Do MH, et al. High-Glucose or -Fructose Diet Cause Changes of the Gut Microbiota and Metabolic Disorders in Mice without Body Weight Change. Nutrients. 2018;10(6):761. 10.3390/nu10060761. 10.3390/nu10060761 PMC6024874 29899272

[66] Kashani A, Brejnrod AD, Jin C, Kern T, Madsen AN, Holm LA, Gerber GK, Holm JC, Hansen T, Holst B, Arumugam M (2019). Impaired glucose metabolism and altered gut microbiome despite calorie restriction of ob/ob mice. Animal Microbiome.

[67] Armougom F, Henry M, Vialettes B, Raccah D, Raoult D (2009). Monitoring bacterial community of human gut microbiota reveals an increase in Lactobacillus in obese patients and Methanogens in anorexic patients. Plos One.

[68] Stsepetova J (2011). Diversity and metabolic impact of intestinal Lactobacillus species in healthy adults and the elderly. Br J Nutr.

[69] Ignacio A (2016). Correlation between body mass index and faecal microbiota from children. Clin Microbiol Infect.

[70] Anhe FF (2020). Type 2 diabetes influences bacterial tissue compartmentalisation in human obesity. Nat Metab.

[71] Massier L, Chakaroun R, Tabei S, Crane A, Didt KD, Fallmann J, von Bergen M, Haange SB, Heyne H, Stumvoll M, Gericke M, Dietrich A, Blüher M, Musat N, Kovacs P (2020). Adipose tissue derived bacteria are associated with inflammation in obesity and type 2 diabetes. Gut.

[72] Hou YP (2017). Human Gut Microbiota Associated with Obesity in Chinese Children and Adolescents. Biomed Res Int.

[73] Qin J, Li Y, Cai Z, Li S, Zhu J, Zhang F, Liang S, Zhang W, Guan Y, Shen D, Peng Y, Zhang D, Jie Z, Wu W, Qin Y, Xue W, Li J, Han L, Lu D, Wu P, Dai Y, Sun X, Li Z, Tang A, Zhong S, Li X, Chen W, Xu R, Wang M, Feng Q, Gong M, Yu J, Zhang Y, Zhang M, Hansen T, Sanchez G, Raes J, Falony G, Okuda S, Almeida M, LeChatelier E, Renault P, Pons N, Batto JM, Zhang Z, Chen H, Yang R, Zheng W, Li S, Yang H, Wang J, Ehrlich SD, Nielsen R, Pedersen O, Kristiansen K, Wang J (2012). A metagenome-wide association study of gut microbiota in type 2 diabetes. Nature.

[74] Dao MC, Everard A, Aron-Wisnewsky J, Sokolovska N, Prifti E, Verger EO, Kayser BD, Levenez F, Chilloux J, Hoyles L, Dumas ME, Rizkalla SW, Doré J, Cani PD, Clément K, MICRO-Obes Consortium (2016). Akkermansia muciniphila and improved metabolic health during a dietary intervention in obesity: relationship with gut microbiome richness and ecology. Gut.

[75] Cani PD, de Vos WM (2017). Next-Generation Beneficial Microbes: The Case of Akkermansia muciniphila. Front Microbiol.

[76] Depommier C, Everard A, Druart C, Plovier H, van Hul M, Vieira-Silva S, Falony G, Raes J, Maiter D, Delzenne NM, de Barsy M, Loumaye A, Hermans MP, Thissen JP, de Vos WM, Cani PD (2019). Supplementation with Akkermansia muciniphila in overweight and obese human volunteers: a proof-of-concept exploratory study. Nat Med.

[77] Cox LM, Sohn J, Tyrrell KL, Citron DM, Lawson PA, Patel NB, Iizumi T, Perez-Perez GI, Goldstein EJC, Blaser MJ (2017). Description of two novel members of the family Erysipelotrichaceae: Ileibacteriumvalens gen. nov., sp. nov. and Dubosiella newyorkensis, gen. nov., sp. nov., from the murine intestine, and emendation to the description of Faecalibacterium rodentium. Int J Syst Evol Microbiol.

[78] Mailing LJ, Allen JM, Buford TW, Fields CJ, Woods JA (2019). Exercise and the Gut Microbiome: A Review of the Evidence, Potential Mechanisms, and Implications for Human Health. Exerc Sport Sci Rev.

