# Supplementary material for: Novel insights into the genetically obese (ob/ob) and diabetic (db/db) mice: two sides of the same coin
Source: Microbiome. 2021 Jun 28;9:147. doi: 10.1186/s40168-021-01097-8 (PMC8240277; doi:10.1186/s40168-021-01097-8)
Supplement: Supplementary file 2 — Additional file 1: Table S1. RT-qPCR primer sequences for the targeted mouse genes. [file 40168_2021_1097_MOESM2_ESM.docx]

| Table S1: RT-qPCR primer sequences for the targeted mouse genes. | | | |
| --- | --- | --- | --- |
| Gene symbol | Protein | Forward Primer sequence (5’-3’) | Reverse Primer sequence (5’-3’) |
| *Abcb11* | BSEP | AGATACAACCGAAGGGGACA | TCAACTTCTTCCACAAGCACA |
| *Abcb4*  *Abcg5*  *Abcg8*  *Acaca*  *Adgre1*  *Baat*  *Ccl2*  *Cd14*  *Cd163*  *Cd36*  *Cd68*  *Cebpa*  *Col1a1*  *Cpt1a*  *Cyp27a1*  *Cyp7a1*  *Cyp8b1*  *Fabp6*  *Fasn*  *Hmgcr*  *Hnf4a*  *Ifng*  *Il1b*  *Il6*  *Itgax*  *Nlrp3*  *Oatp1b2*  *Ppara*  *Pparg*  *Ptgs2*  *Slc10a1*  *Slc10a2*  *Slc27a5*  *Slc51a*  *Slc51b*  *Tgfb1*  *Tlr2*  *Tlr4*  *Tlr5*  *Tnf*  *Rpl19* | MDR2  ABCG5  ABCG8  ACCα  F4/80  BAT  MCP1  CD14  CD163  CD36  CD68  CEBPα  COL1A1  CPT1α  CYP27A1  CYP7A1  CYP8B1  IBABP  FAS  HMGCR  HNF4α  IFNγ  IL1β  IL6  CD11C  NLRP3  OATP1B2  PPARα  PPARγ  PTGS2  NTCP  ASBT  BAL  OSTα  OSTβ  TGFβ  TLR2  TLR4  TLR5  TNFα  RPL19 | GAGCCCGTGCTGTTCTCTAC  ACCTTACCCACGGTTCCTTT  CCGTCGTCAGATTTCCAATGA  GTTGAGACGCTGGTTTGTAGAA  TGACAACCAGACGGCTTGTG  GCACAGGCTCATCAACAAGA  GCAGTTAACGCCCCACTCA  CCTGCCCTCTCCACCTTAGAC  TCCACACGTCCAGAACAGTC  GCCAAGCTATTGCGACATGA  CTTCCCACAGGCAGCACAG  GAGCCGAGATAAAGCCAAACA  CCTCAGGGTATTGCTGGACAAC  AGACCGTGAGGAACTCAAACCTAT  TCTGGCTACCTGCACTTCCT  GGGATTGCTGTGGTAGTGAGC  GATCCGTCGCGGAGATAAGG  CAAGGCTACCGTGAAGATGGA  agatgaaggtggcagaggtg  TGGTGGGACCAACCTTCTAC  AAGAGGTCCATGGTGTTTAAGG  ttcttcagcaacagcaaggc  TCGCTCAGGGTCACAAGAAA  ACAAGTCGGAGGCTTAATTACACAT  ACGTCAGTACAAGGAGATGTTGGA  GCCCAAGGAGGAAGAAGAAG  ATCCCGTGACTAATCCAACA  CAACGGCGTCGAAGACAAA  CTGCTCAAGTATGGTGTCCATGA  TGACCCCCAAGGCTCAAATAT  GGACAAGGTGCCCTACAAAG  TGGGTTTCTTCCTGGCTAGACT  TGTGTGTGAAGGAACCTGGA  TACAAGAACACCCTTTGCCC  GTATTTTCGTGCAGAAGATGCG  AGGACCTGGGTTGGAAGTG  CACCACTGCCCGTAGATGAA  CCCTCAGCACTCTTGATTGC  TCTCCTGACCCTGCTTTGTT  TCGAGTGACAAGCCTGTAGCC  GAAGGTCAAAGGGAATGTGTTCA | TCTGTTTCTGTCCCCCACTC  ACGCATAATCACTGCCTGCT  GGCTTCCGACCCATGAATG  GGTCCTTATTATTGTCCCAGACGTA  GCAGGCGAGGAAAAGATAGTGT  TAGAGCACACCACGTTCCTG  TCCAGCCTACTCATTGGGATCA  TCAGTCCTCTCTCGCCCAAT  CCTTGGAAACAGAGACAGGC  ATCTCAATGTCCGAGACTTTTCAAC  AATGATGAGAGGCAGCAAGAGG  GCGCAGGCGGTCATTG  ACCACTTGATCCAGAAGGACCTT  TGAAGAGTCGCTCCCACT  GTGTGTTGGATGTCGTGTCC  GGTATGGAATCAACCCGTTGTC  CGGGTTGAGGAACCGATCAT  CCCACGACCTCCGAAGTCT  gtagcatgggctgggtgtt  GCCATCACAGTGCCACATAC  ATCGAGGATGCGGATGGA  ACTCCTTTTCCGCTTCCTGA  CATCAGAGGCAAGGAGGAAAAC  TTGCCATTGCACAACTCTTTTC  ATCCTATTGCAGAATGCTTCTTTACC  AGAAGAGACCACGGCAGAAG  ACCAAACTGCTGCTCTATAAACT  TGACGGTCTCCACGGACAT  TGAGATGAGGACTCCATCTTTATTCA  TGAACCCAGGTCCTCGCTTA  ACAGCCACAGAGAGGGAGAA  TGTTCTGCATTCCAGTTTCCAA  ACCCGGACAACTTTGTGAAG  CGAGGAATCCAGAGACCAAA  TTTCTGTTTGCCAGGATGCTC  GTTGGTTGTAGAGGGCAAGG  GCCTCGGAATGCCAGCTT  TGCTTCTGTTCCTGGACCCA  GCCCCCTAGCAGTGAGTT  TTGAGATCCATGCCGTTGG  CCTTGTCTGCCTTCAGCTTGT |
