# Supplementary figures and images for: Novel insights into the genetically obese (ob/ob) and diabetic (db/db) mice: two sides of the same coin
Source: Microbiome. 2021 Jun 28;9:147. doi: 10.1186/s40168-021-01097-8 (PMC8240277; doi:10.1186/s40168-021-01097-8)

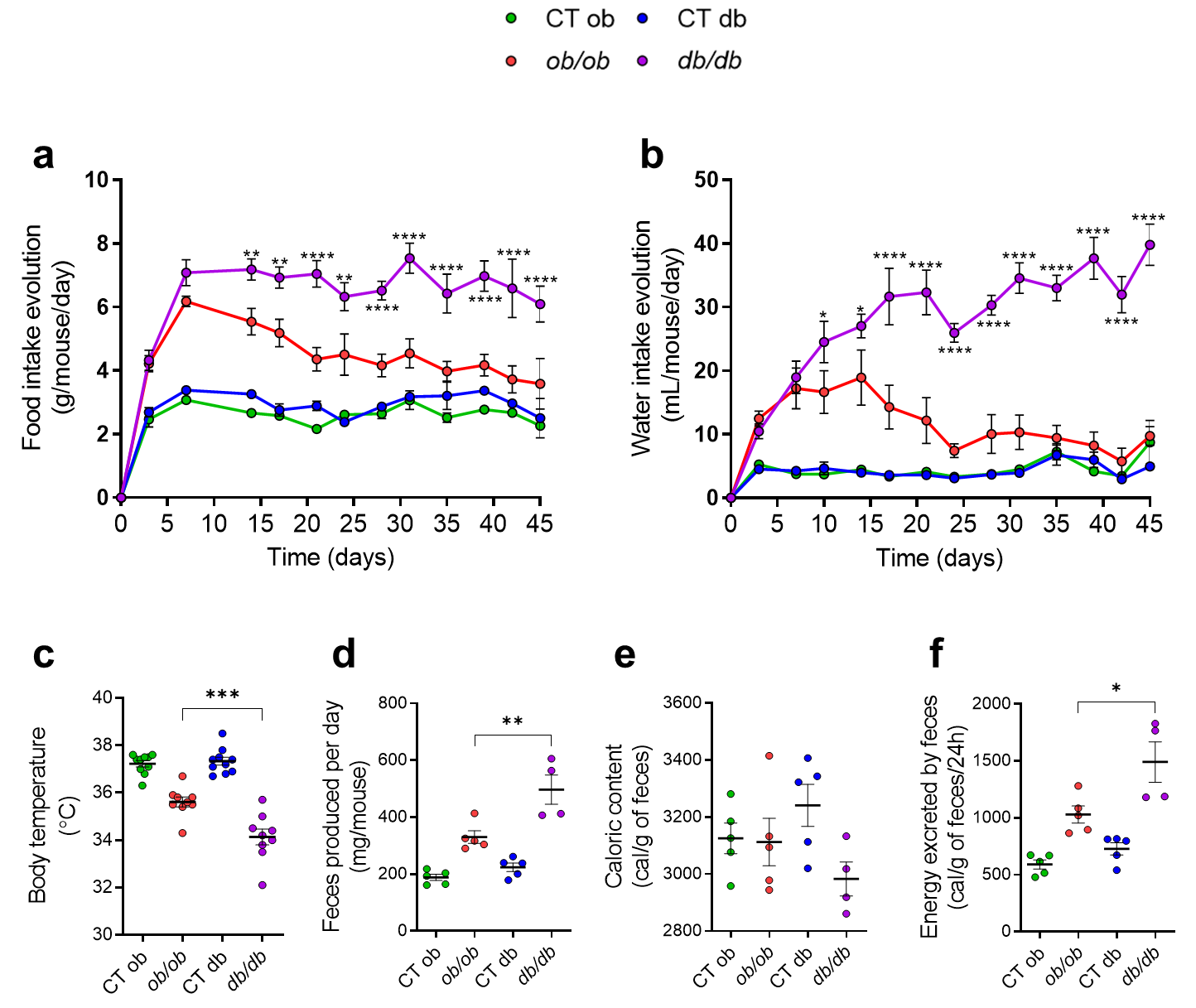

Supplement: Supplementary file 3 — Additional file 2: Fig. S1. Different food intake and water intake profile, body temperature, feces production and energy excreted by feces in ob/ob and db/db mice. (a) Food intake evolution (g/mouse/day) measured for the entire experiment (n = 4-5). (b) Water intake evolution (mL/mouse/day) measured for the entire experiment (n = 4-5). (c) Body temperature (°C) (n = 9-10). (d) Feces produced per day (mg/mouse) (n = 4-5). (e) Caloric content (cal/g of feces) in 24h feces collected (n = 4-5). (f) Energy excreted by feces (cal/g of feces/24h) (n = 4-5). Green: CT ob lean mice, red: ob/ob mice, blue CT db lean mice, and violet: db/db mice. Data are presented as the mean ± s.e.m, *P < 0.05, **P < 0.01, ***P < 0.001, ****P < 0.0001. Data were analyzed by one-way ANOVA followed by Tukey’s post hoc test. [file 40168_2021_1097_MOESM3_ESM.tif]

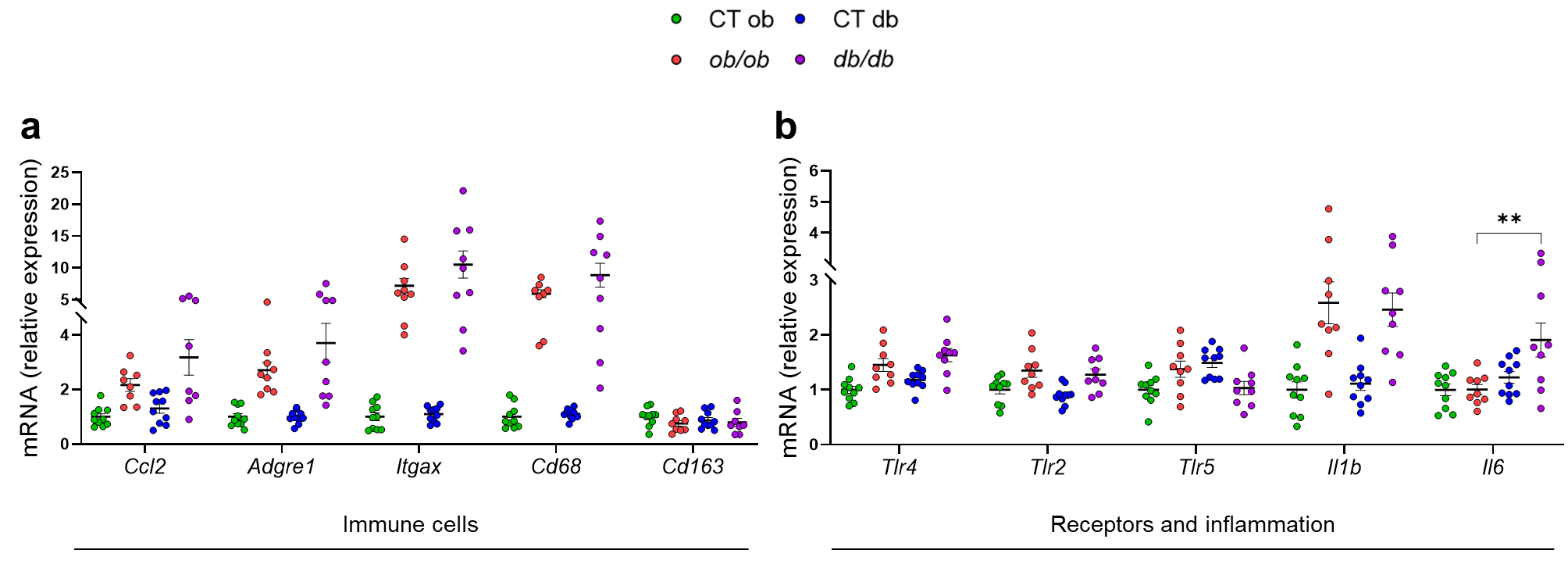

Supplement: Supplementary file 4 — Additional file 3: Fig. S2. Similar visceral adipose tissue features between ob/ob and db/db mice. (a) mRNA expression of VAT immune cells markers measured by RT-qPCR. (b) mRNA expression of VAT receptors and inflammatory cytokines markers measured by RT-qPCR. Green: CT ob lean mice, red: ob/ob mice, blue CT db lean mice, and violet: db/db mice. Data are presented as the mean ± s.e.m., **P < 0.01 (n = 8-10). For the mRNA expression, relative units were calculated versus the mean of the CT ob mice values set at 1. Data were analyzed by one-way ANOVA followed by Tukey’s post hoc test. [file 40168_2021_1097_MOESM4_ESM.tif]

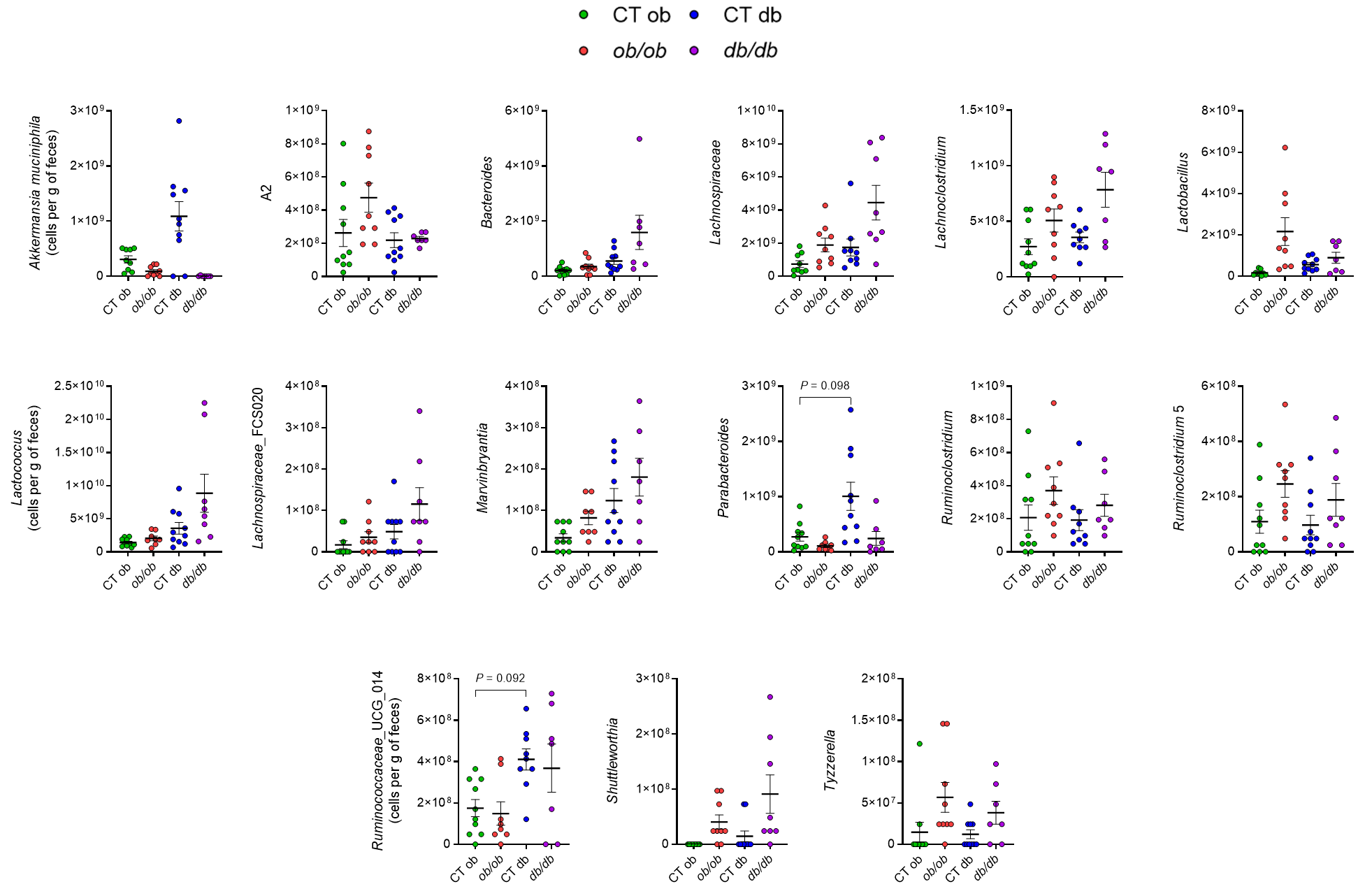

Supplement: Supplementary file 6 — Additional file 5: Fig. S3. Different quantitative gut microbiota profiles among the four genotype groups. Green: CT ob lean mice, red: ob/ob mice, blue CT db lean mice, and violet: db/db mice. Data are presented as the mean ± s.e.m, (n = 7–10). Genera with a prevalence across samples lower than 15% were excluded. Data were analyzed by Kruskal-Wallis test with Dunn’s multiple comparison test. [file 40168_2021_1097_MOESM6_ESM.tif]
