# Supplementary material for: Novel insights into the genetically obese (ob/ob) and diabetic (db/db) mice: two sides of the same coin
Source: Microbiome. 2021 Jun 28;9:147. doi: 10.1186/s40168-021-01097-8 (PMC8240277; doi:10.1186/s40168-021-01097-8)
Supplement: Supplementary file 7 — Additional file 6: Table S3. Taxa-metabolic parameters associations. Spearman correlation between bacterial genera and selected metabolic parameters. Genera whose prevalence was less than 15% of the samples were excluded. Multiple testing correction was performed (Benjamini-Hochberg method). [file 40168_2021_1097_MOESM7_ESM.docx]

| Table S3: Taxa-metabolic parameters associations. | | | | |
| --- | --- | --- | --- | --- |
| Bacteria | Categories | Metabolic parameters | Spearman correlation  (**Day 42**) | |
|  |  |  | rho | FDR  adjusted *p* value |
| *Akkermansia muciniphila* | Organs weight | Vat_weight | -0.617 | 0.007 |
|  |  | Bat_weight | -0.574 | 0.019 |
|  |  | Liver_weight | -0.550 | 0.029 |
|  | Body weight/composition | Final body weight | -0.623 | 0.006 |
|  |  | Final fat mass | -0.660 | 0.002 |
|  | Glucose profile | Area under the curve_ glucose | -0.637 | 0.004 |
|  |  | Area under the curve_ insulin | -0.519 | 0.043 |
|  |  | Insulin resistance index | -0.632 | 0.005 |
|  | Lipid metabolism | Liver_cholesterol content | -0.526 | 0.039 |
|  |  | Liver_*Pparg* mRNA | -0.606 | 0.009 |
|  |  | Liver_*Cpt1a* mRNA | -0.514 | 0.045 |
|  |  | Sat_*Acaca* mRNA | **0.624** | 0.006 |
|  |  | Sat_*Fasn* mRNA | **0.536** | 0.033 |
|  |  | Sat_*Cpt1a* mRNA | -0.570 | 0.019 |
|  | Bile acids metabolism | Liver_*Abcb11* mRNA | **0.545** | 0.030 |
|  |  | Liver_*Abcb4* mRNA | -0.559 | 0.024 |
|  | Inflammation | Liver_*Tlr5* mRNA | -0.509 | 0.049 |
|  |  | Sat_*Ccl2* mRNA | -0.623 | 0.006 |
|  |  | Sat_*Itgax* mRNA | -0.613 | 0.007 |
|  |  | Sat_*Cd68* mRNA | -0.597 | 0.011 |
|  |  | Vat_*Adgre1* mRNA | -0.642 | 0.004 |
|  |  | Vat_*Itgax* mRNA | -0.616 | 0.006 |
| *Bacteroides* | Organs weight | Vat_weight | **0.523** | 0.041 |
|  |  | Sol_weight | -0.545 | 0.029 |
|  | Lipid metabolism | Liver_*Hmgcr* mRNA | -0.524 | 0.041 |
| *Bilophila* | Bile acids metabolism | Ileum_*Fabp6* mRNA | **0.545** | 0.029 |
| *Clostridium_sensu_ stricto*_1 | Organs weight | Sol_weight | **0.599** | 0.010 |
|  | Lipid metabolism | Liver_*Fasn* mRNA | **0.632** | 0.005 |
|  |  | Liver_*Hmgcr* mRNA | **0.821** | < 0.001 |
|  | Inflammation | Liver_*Cd68* mRNA | **0.649** | 0.004 |
|  | Bile acids metabolism | Liver_*Slc27a5* mRNA | -0.647 | 0.004 |
|  |  | Liver_*Hnf4a* mRNA | -0.539 | 0.032 |
| *Dubosiella* | Organs weight | Sol_weight | **0.793** | < 0.001 |
|  | Lipid metabolism | Liver_*Hmgcr* mRNA | **0.662** | 0.003 |
|  | Inflammation | Vat_*Il6* mRNA | -0.524 | 0.040 |
| *Escherichia/Shigella* | Glucose profile | Area under the curve_glucose | **0.583** | 0.015 |
|  | Temperature | Final body temperature | -0.517 | 0.043 |
| *Faecalibaculum* | Lipid metabolism | Liver_*Hmgcr* mRNA | **0.545** | 0.030 |
|  | Bile acids metabolism | Liver_*Slc27a5* mRNA | -0.519 | 0.043 |
|  |  | Liver_*Hnf4a* mRNA | -0.616 | 0.007 |
| *Lachnospiraceae* UCG_006 | Organs weight | Vat_weight | **0.517** | 0.004 |
|  |  | Sol_weight | -0.684 | < 0.001 |
|  | Bile acids metabolism | Liver_*Abcb11* mRNA | -0.530 | 0.036 |
|  | Inflammation | Sat_*Itgax* mRNA | **0.557** | 0.025 |
| *Lactobacillus* | Organs weight | Gas_weight | -0.564 | 0.022 |
|  | Lipid content | Liver_total lipids content | **0.513** | 0.046 |
|  | Bile acids metabolism | Liver_*Slc51b* mRNA | **0.533** | 0.035 |
|  | Inflammation | SAT_*Itgax* mRNA | **0.537** | 0.033 |
|  |  | SAT_*Cd68* mRNA | **0.517** | 0.043 |
|  |  | VAT_*Adgre1* mRNA | **0.501** | 0.049 |
|  |  | VAT_*Itgax* mRNA | **0.544** | 0.030 |
| *Marvinbryantia* | Organs weight | Sol_weight | -0.536 | 0.033 |
|  | Glucose profile | Plasma insulin_-30 minutes | **0.522** | 0.041 |
|  | Inflammation | SAT_*Itgax* mRNA | **0.525** | 0.040 |
| *Olsenella* | Organs weight | Sol_weight | **0.694** | < 0.001 |
| *Parabacteroides* | Inflammation | Liver_*Cd68* mRNA | -0.626 | 0.006 |
|  | Bile acids metabolism | Liver_*Oatp1b2* mRNA | **0.559** | 0.024 |
| Shuttleworthia | Organs weight | Vat_weight | **0.574** | 0.019 |
|  |  | Bat_weight | **0.526** | 0.039 |
|  |  | Gas_weight | -0.645 | 0.004 |
|  |  | Liver_weight | **0.528** | 0.038 |
|  | Lipid metabolism | Sat_*Cpt1a* mRNA | **0.594** | 0.011 |
|  | Bile acids metabolism | Liver_*Abcb11* mRNA | -0.563 | 0.022 |
|  | Inflammation | Sat_*Ccl2* mRNA | **0.571** | 0.019 |
|  |  | Sat_*Itgax* mRNA | **0.621** | 0.006 |
|  |  | Sat_*Cd68* mRNA | **0.593** | 0.011 |
|  |  | Vat_*Itgax* mRNA | **0.639** | 0.004 |
| *Turicibacter* | Organs weight | Sol_weight | **0.675** | 0.002 |
|  | Lipid metabolism | Liver_*Fasn* mRNA | **0.647** | 0.004 |
|  |  | Liver_*Hmgcr* mRNA | **0.709** | < 0.001 |
|  | Inflammation | Liver_*Cd68* mRNA | **0.623** | 0.006 |
|  | Bile acids metabolism | Liver_*Slc27a5* mRNA | -0.571 | 0.019 |
| *Tyzzerella* | Body composition | Final fat mass | **0.513** | 0.046 |
|  | Organs weight | Bat_weight | **0.542** | 0.031 |
|  |  | Gas_weight | -0.571 | 0.019 |
|  | Bile acids metabolism | Liver_*Ntcp* mRNA | -0.569 | 0.019 |
|  |  | Ileum_*Slc51b* mRNA | **0.518** | 0.043 |
|  | Inflammation | Sat_*Itgax* mRNA | **0.531** | 0.031 |
